# Induced pluripotent stem cell-derived neuronal cells from a sporadic Alzheimer’s disease donor as a model for investigating AD-associated gene regulatory networks

**DOI:** 10.1186/s12864-015-1262-5

**Published:** 2015-02-14

**Authors:** Amir M Hossini, Matthias Megges, Alessandro Prigione, Bjoern Lichtner, Mohammad R Toliat, Wasco Wruck, Friederike Schröter, Peter Nuernberg, Hartmut Kroll, Eugenia Makrantonaki, Christos C Zoubouliss, James Adjaye

**Affiliations:** Departments of Dermatology, Venereology, Allergology and Immunology, Dessau Medical Center, 06847 Dessau, Germany; Institute for Stem Cell Research and Regenerative Medicine, Heinrich Heine University Duesseldorf, Moorenstr. 5, 40225 Duesseldorf, Germany; Cologne Center for Genomics (CCG), Institute for Genetics, University of Cologne, 50931 Cologne, Germany; Institute for Transfusion Medicine Dessau, Red Cross Blood Transfusion Service NSTOB, 06847 Dessau, Germany; Molecular Embryology and Aging Group, Department of Vertebrate Genomics, Max Planck Institute for Molecular Genetics, 14195 Berlin, Germany; Department of Biology, Chemistry and Pharmacy, Institute of Chemistry and Biochemistry, Freie Universität Berlin, Thielallee 63, 14195 Berlin, Germany; Geriatrics Research Group, Department of Geriatric Medicine, Charité Universitätsmedizin Berlin, Reinickendorfer Str. 61, 13447 Berlin, Germany; Current address: Max Delbrueck Center for Molecular Medicine (MDC), Robert Roessle Str. 10, D-13125 Berlin, Germany

**Keywords:** Alzheimer’s disease, Skin cells, γ-secretase, Induced pluripotent stem cells, *TNFRSF1A*, Neuronal cells, p-tau, Transcriptome, Proteasome

## Abstract

**Background:**

Alzheimer’s disease (AD) is a complex, irreversible neurodegenerative disorder. At present there are neither reliable markers to diagnose AD at an early stage nor therapy. To investigate underlying disease mechanisms, induced pluripotent stem cells (iPSCs) allow the generation of patient-derived neuronal cells in a dish.

**Results:**

In this study, employing iPS technology, we derived and characterized iPSCs from dermal fibroblasts of an 82-year-old female patient affected by sporadic AD. The AD-iPSCs were differentiated into neuronal cells, in order to generate disease-specific protein association networks modeling the molecular pathology on the transcriptome level of AD, to analyse the reflection of the disease phenotype in gene expression in AD-iPS neuronal cells, in particular in the ubiquitin-proteasome system (UPS), and to address expression of typical AD proteins.

We detected the expression of p-tau and GSK3B, a physiological kinase of tau, in neuronal cells derived from AD-iPSCs. Treatment of neuronal cells differentiated from AD-iPSCs with an inhibitor of γ-secretase resulted in the down-regulation of p-tau. Transcriptome analysis of AD-iPS derived neuronal cells revealed significant changes in the expression of genes associated with AD and with the constitutive as well as the inducible subunits of the proteasome complex. The neuronal cells expressed numerous genes associated with sub-regions within the brain thus suggesting the usefulness of our *in-vitro* model. Moreover, an AD-related protein interaction network composed of APP and GSK3B among others could be generated using neuronal cells differentiated from two AD-iPS cell lines.

**Conclusions:**

Our study demonstrates how an iPSC-based model system could represent (i) a tool to study the underlying molecular basis of sporadic AD, (ii) a platform for drug screening and toxicology studies which might unveil novel therapeutic avenues for this debilitating neuronal disorder.

**Electronic supplementary material:**

The online version of this article (doi:10.1186/s12864-015-1262-5) contains supplementary material, which is available to authorized users.

## Background

Alzheimer’s disease (AD) is characterized by histopathological changes, designated as senile plaques and fibrillary deposits, which ultimately lead to the death of neuronal cells in particular in the cerebral cortex of the brain [1,2]. The familial form of AD is rare, affecting less than five percent of AD patients and has been associated with mutations of *Presenilin 1* (*PSEN1*), *Presenilin 2* (*PSEN2*) and *Amyloid Precursor Protein* (*APP*) [3,4]. These mutations result in incorrect cleavage of the protein, producing a deposited protein of amyloid-β (Aβ) that is more likely to form plaques [1,5]. Little is known about the molecular basis of multifactorial sporadic AD. In post-mortem examination of patients with AD, massive accumulation of two types of amyloid fibril senile plaques (Aβ40, Aβ42) and hyperphosphorylated tau forming paired helical filaments could be detected [6,7]. Both types of amyloid fibrils are mainly created enzymatically by β- and γ-secretase activity from the APP [8].

The most widely accepted theory for the onset of sporadic AD is the accumulation of extracellular Aβ42 in an aggregated state in the brain, subsequently leading to the formation of neurofibrillary tangles (NFT) containing hyperphosphorylated tau proteins and consequently to its inactivation, thus leading to inhibition of binding to the spindle apparatus and hence disrupted axonal transport [9,10]. The major modification of tau is its phosphorylation. Its hyperphosphorylation has been shown to be the critical step in the formation of NFTs [11,12]. One of the kinases that phosphorylates tau *in-vivo* is glycogen synthase kinase-3β (GSK3B), which is widely expressed in all tissues with elevated expression in developing brains [13]. Unlike many other kinases, GSK3B is believed to be permanently active in resting cells and in neurons without extracellular stimulation and can be inactivated by Ser9 phosphorylation [14].

Moreover, the ubiquitin-proteasome system (UPS) has been shown to be involved in the pathogenesis of AD [15-18].

The UPS consists of the 26S proteasome and the small protein ubiquitin, a post-translational modification, and is operative in all eukaryotes for intracellular protein homeostasis and quality [19,20]. The alternative form of the constitutive proteasome is the immunoproteasome complex [21]. It was demonstrated in *in-vitro* experiments that the accumulation of Aβ peptide in *APP*/*PSEN1* mutant neuronal cell culture leads to the inhibition of the proteasome as well as the de-ubiquitinating enzymes (DUBs) [15]. Despite increasing knowledge on AD-associated pathology, the molecular mechanisms underlying the cause of sporadic and familial AD are still not completely understood. This limitation is primarily due to limited access and availability of viable neuronal cells from AD patients because of ethical and practical reasons. Human induced pluripotent stem (iPSCs) cells enables the generation of clinically relevant neuronal cells *in-vitro*. Patient-derived skin cells are an easily accessible source for reprogramming and the obtained neuronal cells can be used to investigate the pathogenesis of neuronal disorders, including Parkinson disease and Alzheimer’s disease [22-26].

In our study, we reprogrammed dermal fibroblasts obtained from an 82-year-old female patient diagnosed with late-stage AD to iPSCs in one reprogramming experiment. The two derived iPSC lines showed pluripotency-associated properties similar to human embryonic stem cells (hESC) and they could be successfully differentiated into neuronal cells *in-vitro*. The differentiated neuronal cells seemed to reflect the sporadic AD phenotype in the brain of the patient, including the expression of p-tau proteins, the up-regulation of GSK3B protein and its phosphorylation in contrast to the parental dermal fibroblast cells. In addition, numerous AD-related genes were found to be down-regulated, as revealed by microarray-based gene expression analysis of one neuronal differentiation experiment per AD-iPS cell line. Most notably these genes could be allocated to brain regions affected by Alzheimer’s disease.

We demonstrated a down-regulation of p-tau proteins in AD neuronal cells with an inhibitor of γ-secretase. In addition, neuronal cells differentiated from the patient iPSCs showed an up-regulation of a number of neuronal and biological processes, which include development of the nervous system, neurogenesis, WNT signaling pathway, the lysosome, glutathion metabolism as well as the alanine, aspartate and glutamate metabolism. Furthermore, the down-regulation of AD-related genes enabled us to successfully construct a protein association network using the STRING database reflecting the presence of AD-related disease mechanisms in our iPSCs model. Finally, we could show that gene regulation of the constitutive as well as of the inducible subunits of the proteasome complex is affected in iPSC-derived neurons from the AD patient compared to the healthy subject. Further investigations are needed to better understand the molecular basis of the onset and progression of Alzheimer’s disease. Elucidating the molecular mechanism of sporadic AD by modeling it via iPSC technology and protein association networks could provide valuable information needed to uncover appropriate strategies against the early onset of the disease.

## Results

### Generation and characterization of sporadic AD-iPSCs

Dermal fibroblasts were isolated from an 82-year-old woman diagnosed with final stage AD. The cell line was named NFH-46, and lack of AD-related mutations, such as *APP*, *PSEN1* and *PSEN2* [1,5], was confirmed by direct sequencing analysis (Additional file 1). HLA haplotype analysis in the AD donor did not reveal any association of HLA alleles to Morbus Alzheimer. The HLA-alleles HLA-A*01:01,*03:01; B*08,*35, C*04:01,*07:01, DRB1*03:01,*11:01 were found in NFH-46. However, the Alzheimer-related HLA-alleles HLA-A*02, HLA-B*07 and HLA-C*07:02 could not be detected.

**Figure 1:**
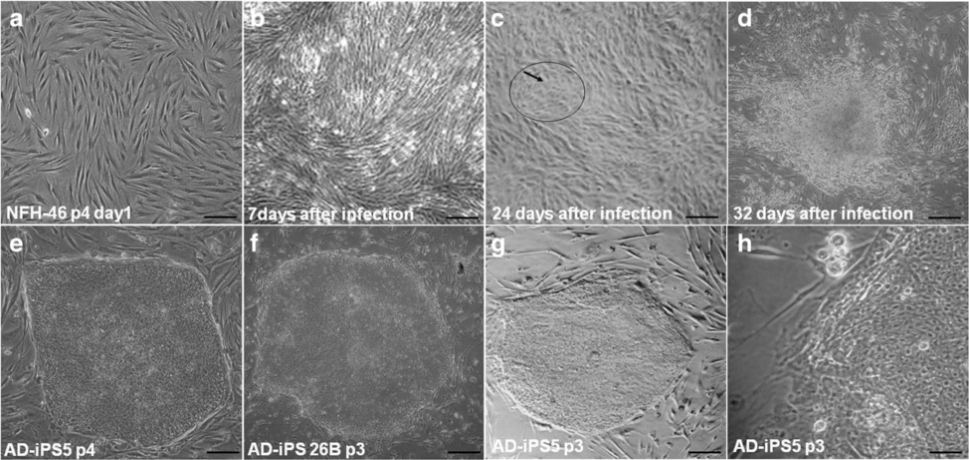
**Generation of human iPSCs from skin fibroblasts of a sporadic Alzheimer patient. (a)**: Morphology of fibroblasts NFH-46 in passage 4 (p4) before viral transduction. **(b)**: Changes in morphology of NFH-46 seven days after infection with retroviruses. **(c)**: Changes of NFH-46 on day 24 after infection shown in circle with arrow. **(d)**: Typical image of non-embryonic stem cell like colony. **(e, f)**: Typical morphology of AD-iPS colonies (AD-iPS-5, passage 4; AD-iPS-26B, passage 3) of one reprogramming experiment. **(g)**: Typical morphology of AD-iPS colony in passage 3(p3). **(h)**: AD-iPSC structure in high magnification. Scale bar, 100 μm.

**Figure 2:**
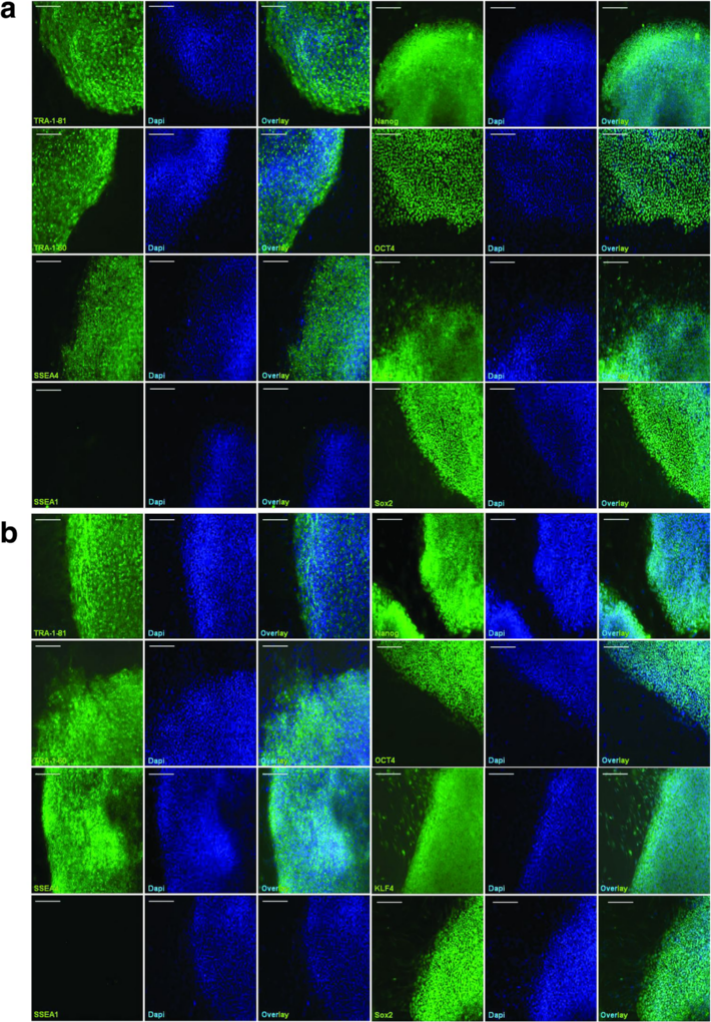
**AD-iPSCs express key pluripotency-associated proteins.** Two AD-iPSC lines were successfully generated with one reprogramming experiment: AD-iPS5 **(a)** and AD-iPS26B **(b)**. Both lines exhibited hESC-like morphologies, were positive for pluripotency-associated marker proteins, such as TRA-1-81, TRA-1-60, SSEA4, and NANOG, and were negative for the differentiation-specific marker SSEA1. Scale bar, 100 μm.

AD-iPSCs were generated by retroviral transduction using the classical Yamanaka cocktail [27], which includes the four transcription factors OCT4, KLF4, SOX2, and c-MYC, as demonstrated previously [28]. In a single reprogramming experiment several colonies exhibiting hESC-like morphologies were identified and manually picked for expansion and characterization. Two iPSC lines, AD-iPS5 and AD-iPS26B, were successfully established from this reprogramming experiment and characterized with respect to pluripotency-associated properties. Both lines exhibited hESC-like morphologies (Figure 1), telomerase activity (Additional file 2), alkaline phosphatase (AP) activity (Additional file 3a), expression of pluripotency-associated markers NANOG, SSEA4, TRA-1-60, and TRA-1-81 (Figure 2), expression of pluripotency-associated genes such as *NANOG*, *POU5F1*, *SOX2*, *LIN28*, *TDGF1*, *DPPA4*, *FGF4*, *GDF3*, *LEFTY1*, *LEFTY2* (Additional file 4) and the genetic fingerprinting pattern of the parental NFH-46 fibroblasts (Additional file 3b).

Finally, the transcriptomes of the AD-iPSC lines are similar to hESCs (H1 and H9) and to iPS lines previously generated from control NFH-2 fibroblasts [28] (Additional file 5).

The ability to differentiate into almost all tissue types as a hallmark of human pluripotent stem cells was analyzed employing embryoid bodies (EBs) based differentiation *in-vitro* and teratoma formation *in-vivo*. The AD-iPSC lines were able to differentiate *in-vitro* into all three embryonic germ layers, as detected by the expression of marker proteins specific for ectoderm (b-TUBULIN III and NESTIN), for mesoderm (Smooth Muscle Actin (SMA) and T/Brachyury), and endoderm (Alpha feto protein (AFP) and SOX17) (Additional file 6).

Finally, both AD-iPSC lines successfully generated teratomas (Additional file 7). For AD-iPS5, the presence of known endoderm-associated structures appeared unclear. However, this must not necessarily imply an impairment of this line towards endoderm differentiation *in-vivo*, since the teratoma assay itself is not standardized [29]. Moreover, in the *in-vitro* differentiated cells from AD-iPS5, SOX17 and AFP, both protein markers representative of endoderm, could be detected. Thus, we consider AD-iPS5 to be pluripotent.

Chromosomal analysis of AD-iPS5 revealed the loss of a gonosome, probably the X chromosome, because during mitosis they revealed a normal female karyotype. This is in agreement with our previous study showing that iPSC lines generated from old donors are more likely to contain chromosomal aberrations [28]. In nine mitoses, a very small supernumerary marker chromosome (sSMC) was found besides the monosomy X (Additional file 8). It is unlikely that the presence of a small supernumaray maker chromosome has an effect on AD-iPS5 as sSMCs are a common phenomenon in human. The karyotypes of the second iPSC line AD-iPS26B and the parental cells NFH-46 were normal (Additional file 8).

### Generation of neuronal cells from AD-iPSCs (AD-iPS neurons)

**Figure 3:**
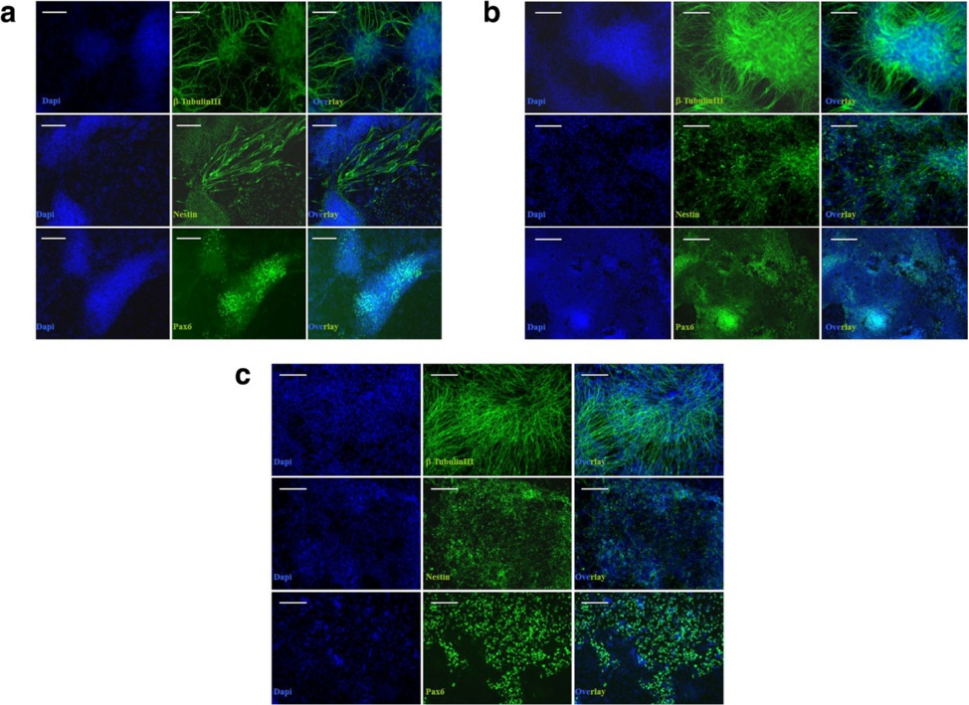
**Neuronal differentiation of AD-iPSCs.** Induction of neuronal cells by simultaneous treatment with inhibitors of transforming growth factor (TGF)-β receptor and MEK1/2 for 4 weeks. Neuronal cells derived from AD-iPSCs **(a)**: AD-iPS5 neurons and **(b)**: AD-iPS26B neurons showed the expression of neuronal markers, including PAX6, NESTIN and -β-TUBULIN III in a similar fashion as neuronal cells derived from the hESC line **(c)**: H9. The neuronal differentiation was carried out once per AD-iPS cell line or H9 cell line. Scale bar, 100 μm.

**Figure 4:**
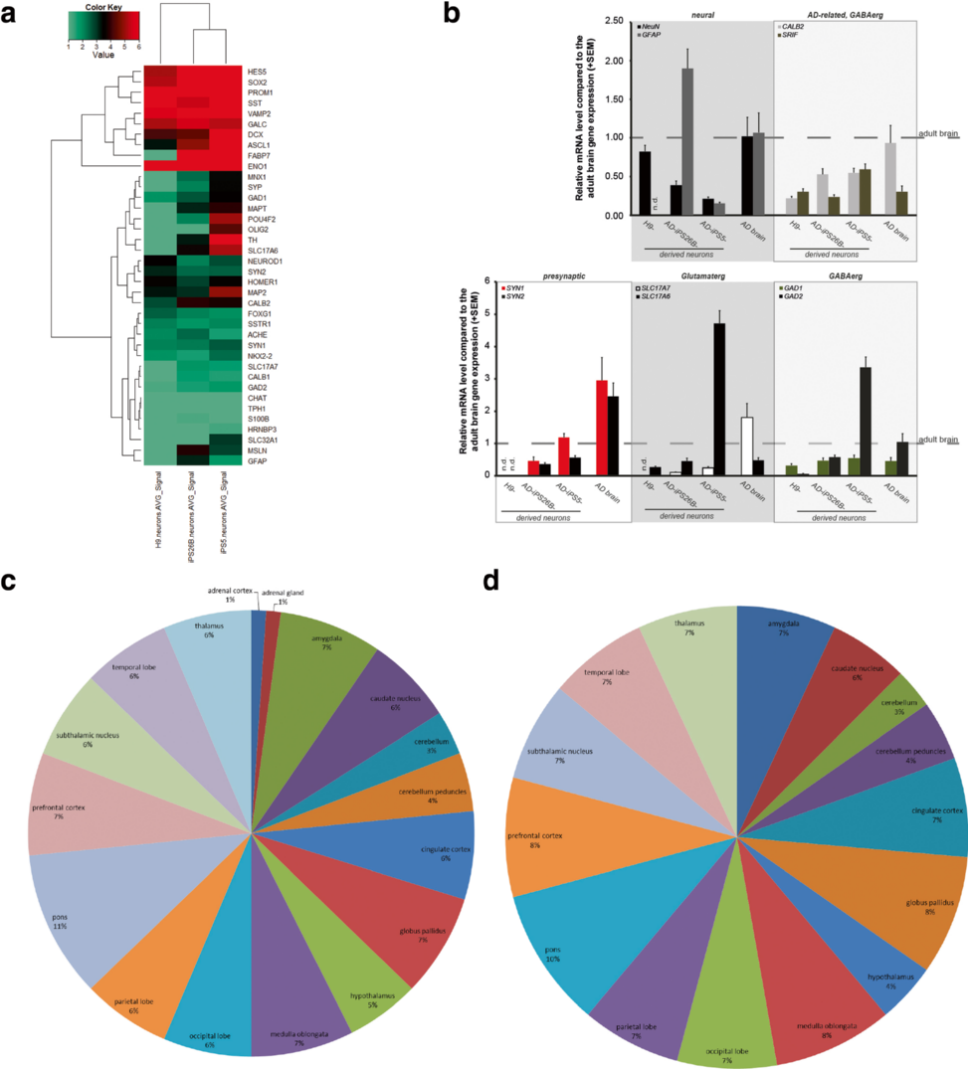
**Expression of neuronal marker genes and genes associated with Alzheimer-related brain regions in AD-iPS neurons. (a)** Cluster analysis of neuronal marker genes in AD-iPS neurons and H9 neurons based on Euclidean distance of microarray-based gene expression values. **(b)** A number of genes of the heatmap data in **(a)** were confirmed by real- time PCR analysis. Bars indicate the RNA level normalized to β-ACTIN first and compared to gene expression of adult brain tissue (non-diseased, male, 21 years; Amsbio). The samples of AD-iPS neurons and H9 neurons consisted of cRNA generated from RNA isolated from a single well of a single neuronal differentiation respectively. In addition, to our data we analyzed AD brain RNA (male, 87 years, diagnosed AD; Amsbio). **(c)** Brain regions associated with genes from the AD KEGG pathway (but neither in Huntington disease nor in Parkinson disease) down-regulated in AD-iPS5 compared to H9 neurons. **(d)** Brain regions associated with genes from the AD KEGG pathway (but neither in Huntington nor in Parkinson disease) down-regulated in AD-iPS26B compared to H9 neurons.

We derived neuronal cells from AD-iPSCs in one experiment to address the potential of these to reflect neuropathological features found in neuronal cells of sporadic AD patients. As a control, neuronal cells were derived from the female hESC line H9 in one differentiation experiment. The neuronal cells were generated following a recently published protocol, which requires the exposure to TGF-β receptor (SB431542) and MEK1/2 (PD0325901) inhibitors [30]. AD-iPSC lines (AD-iPS5 and AD-iPS26B) and H9 were successfully differentiated into neuronal cells. The efficiency of differentiation varied, as AD-iPS5 showed more pronounced neuronal differentiation than AD-iPS26B. All induced neuronal cells were positive for neuronal cell markers PAX6, NESTIN, and b-TUBULIN III as shown in Figure 3. Most of the neuronal marker genes in the heatmap shown in Figure 4a are expressed in a similar manner in AD-iPSC neurons and H9 neurons, hence confirming a neuronal differentiation of comparable quality across all used pluripotent cell lines.

### Expression of neuronal marker genes in AD-iPS neurons and H9 neurons

The heatmap in Figure 4a shows the expression pattern of pre-synaptic and post-synaptic genes as well as markers of distinct subtypes of neural progenitors and mature neuronal cell types in AD-iPS neurons and H9 neurons of one differentiation experiment conducted. Neuronal markers *FABP7*, *HES5*, *SOX2*, *PROM1* and *ASCL1* are expressed in AD-iPS5 and AD-iPS26B neurons, however, *FABP7* was not detected in H9 neurons. *GALC*, a marker of oligodendrocyte progenitor cells is expressed in all samples but lower in AD-iPS26B and H9 neurons. *MAP2*, a marker of neuronal dendrites is expressed in AD-iPS5 neurons but not in neuronal cells of AD-iPS26B and H9. Moreover, markers of retinal ganglion cells (*POU4F2*), dopaminergic neurons (*TH*) and glutamatergic neurons (*SLC17A6*) are expressed in AD-iPS5 neurons (Figure 4a). In addition, neural genes such as *NeuN* (*HRNBP3* or *FOX1*), *GFAP* and *GAD1*, *GAD2* (GABA-ergic genes) on the one hand and specific AD-related neuronal genes such as *CALBINDIN1* and *2* (also known as calretinin) as well as *SST* (somatostatin or *SRIF*) on the other were analyzed. SRIF-positive interneurons are inhibitory neurons which express *GAD1* and/or *GAD2* as well as *CALB2* [31] and are the most affected subtypes of neurons in AD [32]. To confirm the array-derived heatmap data we analyzed relative gene expression by real-time PCR (Figure 4b) using the samples of the same neuronal differentiation experiment which were hybridized for transcriptome analysis. By matching the gene expression to adult brain RNA, again the H9-derived neuronal cells did not express subtype-specific neuronal genes. The astrocyte-specific gene *GFAP* was barely detected in all neural cells compared to the mRNA level of the adult and AD brain (Figure 4b). The neuronal cells from AD-iPS5 and AD-iPS26B were positive for *SYNAPSIN I*, *vGLUT2* (*SLC17A6*) and *GAD2* and have the same transcript level as the AD brain for *CALB2* and *GAD1* (Figure 4b).

### Proof-of-principle drug discovery using sporadic AD-iPSC derived neuronal cells

**Figure 5:**
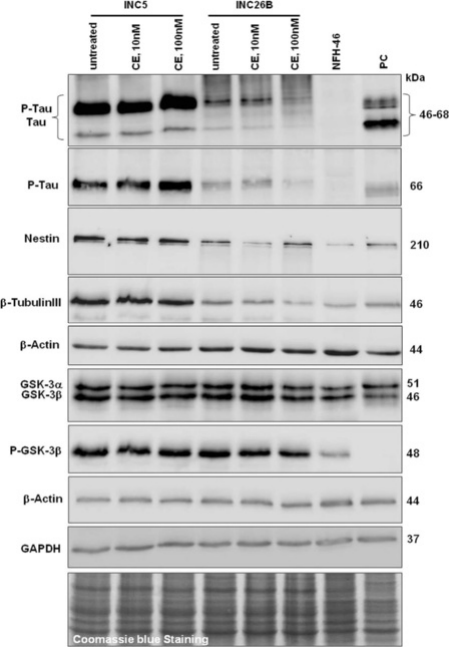
**Treatment of AD neuronal cells with the γ-secretase-inhibitor compound E (CE).** Western blot analysis was used to monitor the expression of phosphorylated tau (p-tau), non-phosphorylated tau, GSK3α/β and phosphorylated GSK3β in AD-iPS5 neurons (INC5) and AD-iPS26B neurons (INC26B). The samples were derived from one well of a single neuronal differentiation. The parental fibroblast cells (NFH-46) were included as control in addition to the positive control (PC) for p-tau represented by lysate from neuroblastoma. β-ACTIN and GAPDH and coomassie blue staining were used to confirm similar protein loading across samples.

In addition to gene expression based analysis of Alzheimer-related genes we evaluated the possible medical relevance of our neuronal cell model in terms of drug discovery and selection of an appropriate therapy for sporadic AD. For this purpose, we subjected the induced neuronal cells to treatment with the γ-secretase-inhibitor Compound E (CE). The experiment was carried out once. Two distinct concentrations were employed: low 10 nM and high 100 nM. After one week of treatment, cells were lysed directly and the protein expression levels of p-tau, tau, p-GSK3B and GSK3B were investigated isolating samples from one well of one inhibitor treatment experiment conducted. Neuronal cells derived from both AD-iPSC lines (AD-iPS5 and AD-iPS26B) exhibited the expression of tau and p-tau, which were undetectable in the parental fibroblasts (NFH-46) (Figure 5). The results were confirmed using two antibodies, one recognizing only p-tau and the other binding to both the phosphorylated and non-phosphorylated forms of tau (Figure 5). Drug treatment did not result in any reduction of p-tau in AD-iPS5 derived neuronal cells. On the other hand, we observed a significant reduction of p-tau and tau expression in neuronal cells differentiated from AD-iPS26B compared to untreated cells following high doses of CE (Figure 5). The expression of both p-GSK3B and GSK3B was significantly higher in neuronal cells compared to parental fibroblasts NFH-46 (Figure 5). However, no evident change in their expression could be identified following CE treatment. Neuronal cells were identified based on the expression of NESTIN and b-TUBULIN III, however, expression was also detected in their parental fibroblast cells, thus confirming previous observations in fibroblasts [33].

### Differential gene expression associated with Alzheimer-related pathways and biological processes in AD-iPSC neurons compared to H9 neurons

Using microarray based gene expression analysis we looked at the changes in the biological processes within the AD-iPS neuronal cells compared to H9 neurons as control. The hybridized samples were isolated from one neuronal differentiation experiment. Processes related to WNT signaling pathway and the alanine, aspartate and glutamate metabolism, in the case of AD-iPS5 neurons as well as the lysosome pathway and glutathion metabolism in the case of AD-iPS26B appeared to be up-regulated compared to H9 neurons. Pathways related to Alzheimer’s disease, Huntington’s disease, Parkinson’s disease and the proteasome were down-regulated in both AD-iPS neurons compared to H9 neurons (Additional files 9 and 10). AD-iPS5 and AD-iPS26B neurons showed up-regulated gene expression for biological processes such as neuronal fate commitment, neuron maturation, response to oxygen radical and/or response to reactive oxygen species (Additional file 9). In contrast to that, the UPS, apoptosis, and oxidative phosphorylation emerged as down-regulated biological processes (Additional file 10). Overall, these data suggest that AD neuronal cells exhibit alterations in key signaling pathways related to cell death, anabolism and catabolism in comparison to the healthy control.

### AD-iPSC neurons show a distinct gene expression pattern of Alzheimer-associated genes of genome wide association studies compared to H9 neurons

**Figure 6:**
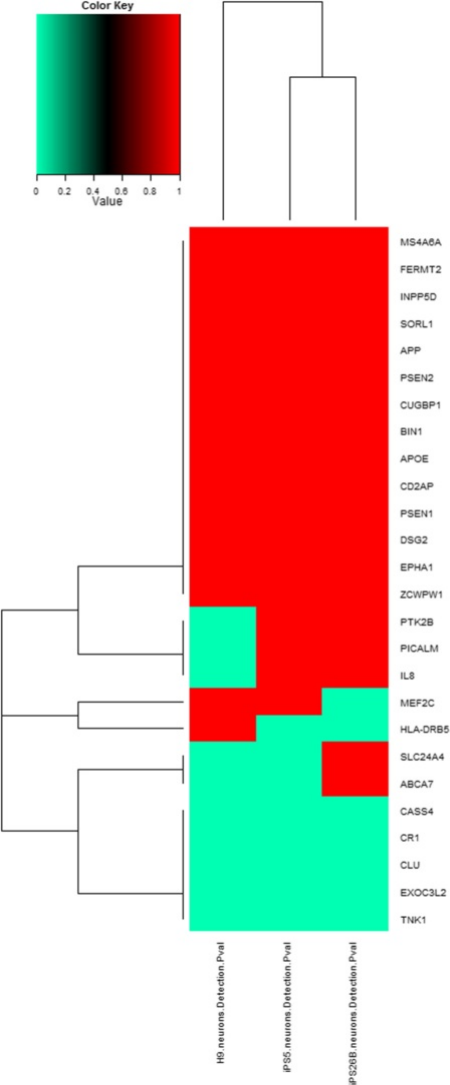
**Expression of Alzheimer risk genes in AD-iPSC derived neurons.** Cluster analysis of Alzheimer risk genes in experiments AD-iPS5 neurons, iPS26B neurons and H9 neurons of one neuronal differentiation experiment each. Up and down-regulated transcripts are depicted in red and green, respectively. RNA from AD-iPS5 neurons, AD-iPS26B neurons and H9 neurons was hybridized onto an Illumina human-8 BeadChip version 3. Alzheimer-associated genes known from genome wide association studies were filtered from the microarray experiments of AD-iPS5 neurons, AD-iPS26B neurons and H9 neurons. Illumina detection p-values were mapped to a binary scale (0 = not expressed if p-value > 0.05, 1 = expressed if p-value < = 0.05). These values were clustered via the R heatmap2 function using Euclidean distance as distance measure.

To further analyze the reflection of Alzheimer-specific gene expression patterns in our iPSC-based model system we performed data mining to extract disease relevant gene expression using an Alzheimer gene list recently published by the European Alzheimer’s Disease Initiative (EADI) [34]. The cluster analysis in Figure 6 showed that AD-iPS5 neurons and AD-iPS26 neurons were more similar to each other than to H9 neurons. Basically there are six gene clusters: (i) a cluster of genes expressed in all experiments such as *APOE* and *APP*, (ii) a cluster of genes expressed in no experiment such as *CASS4* and *CR1*, (iii) a cluster of genes expressed in both AD-iPSC neuron experiments but not in the H9 neuron experiment such as *PTK2B* and *PICALM*, (iv) a singleton cluster of *HLA-DRB5* not expressed in both AD-iPS neuron experiments but expressed in the H9 neurons, (v) a singleton cluster of *MEF2C* not expressed in AD-iPS26B experiments but expressed in AD-iPS5 and the H9 neurons and (vi) a cluster of genes expressed in AD-iPS26B neurons but not in AD-iPS5 and H9 neurons containing genes such as *SLC24A4* and *ABCA7*.

### AD-iPSC neurons show down-regulation of genes involved in Alzheimer’s, Huntington’s and Parkinson’s disease compared to H9 neurons

**Figure 7:**
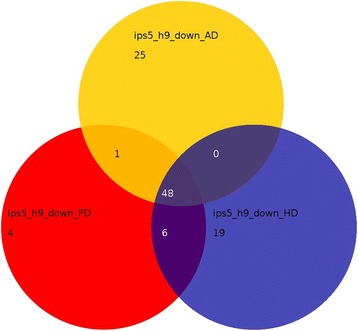
**Gene expression associated with Alzheimer, Parkinson and Huntington disease in AD-iPS5 neurons.** Venn diagram of down-regulated genes in the comparison AD-iPS5 vs. H9 neurons in KEGG pathways Alzheimer disease (AD), Parkinson disease (PD) and Huntington disease (HD). RNA from AD-iPS5 neurons and H9 neurons of one neuronal differentiation experiment was hybridized onto an Illumina human-8 BeadChip version 3. Functional annotation of significantly down-regulated genes from the experiments AD-iPS5 neurons vs. H9 neurons was performed with the DAVID Bioinformatics Resources 6.7. ips5 vs. H9: numbers of significant genes from KEGG pathways for ALzheimer, Parkinsons and Huntington.

**Figure 8:**
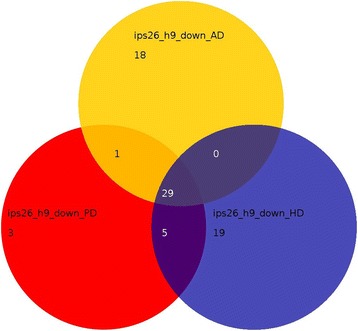
**Gene expression associated with Alzheimer, Parkinson and Huntington disease in AD-iPS26B neurons.** Venn diagram of down-regulated genes in the comparison AD-iPS26B vs. H9 neurons in KEGG pathways Alzheimer disease (AD), Parkinson disease (PD) and Huntington disease (HD). RNA from AD-iPS26B neurons and H9 neurons was hybridized onto an Illumina human-8 BeadChip version 3. Functional annotation of significantly down-regulated genes from the experiments AD-iPS26B neurons vs. H9 neurons was performed with the DAVID Bioinformatics Resources 6.7. ips26 vs. H9: numbers of significant genes from KEGG pathways for ALzheimer, Parkinsons and Huntington.

Analysis of differences in the iPS-derived neurons when compared to the annotations Alzheimer’s disease, Parkinson’s disease and Huntington’s disease revealed that most genes down-regulated in AD-iPS5 vs. H9 neurons (Figure 7) and AD-iPS26B vs. H9 neurons (Figure 8) were common to all three neural disorders. Exclusively associated with Alzheimer’s disease were 16 genes in AD-iPS5 vs. H9 neurons and 10 genes in in AD-iPS26B vs. H9 neurons. These genes were *APP*, *APOE*, *PSENEN*, *CDK5*, *HSD17B10*, *TNFRSF1A*, *PPP3CB*, *PPP3CC*, *CHP*, *GAPDH*, *CAPN2*, *CAPN1*, *ATP2A2*, *GSK3B*, *CALM3* and *CALM2* in the experiment AD-iPS5 vs. H9 neurons and *APP*, *CDK5*, *HSD17B10*, *CHP*, *GAPDH*, *NAE1*, *ATP2A2*, *GSK3B*, *CALM3* and *CALM2* in the experiment AD-iPS26B vs. H9 neurons.

### Brain allocation of Alzheimer-specific genes down-regulated in AD-iPSC neurons compared to H9 neurons

The expression in different brain regions of the Alzheimer-exclusive genes that were found to be down-regulated in AD-iPSC neurons of one differentiation experiment were investigated using the GNF/Atlas organism part. The expression of the largest set of genes was allocated to pons with 12% in the case of AD-iPS5 neurons for the genes *APOE*, *APP*, *ATP2A2*, *CALM2*, *CALM3*, *CAPN2*, *CDK5*, *GAPDH*, *GSK3B* and *PPP3CB* (Figure 4c) and 13% in AD-iPS26B neurons for the genes *APP*, *ATP2A2*, *CALM2*, *CALM3*, *CDK5*, *GAPDH* and *GSK3B* (Figure 4d). This was followed by globus pallidus with 9% in AD-iPS5 neurons for the genes *APP*, *ATP2A2*, *CALM2*, *CALM3*, *CDK5*, *GAPDH* and *PPP3CB* and 9% in AD-iPS26B neurons for the genes *APP*, *ATP2A2*, *CALM2*, *CALM3*, *CDK5* and *GAPDH*. The percentages for medulla oblongata, prefrontal cortex and amygdala were found to be 5–8% (Figure 4c and d).

### An Alzheimer-relevant functional protein association network can be built using an Alzheimer-specific gene set down-regulated in AD-iPSC neurons compared to H9 neurons

**Figure 9:**
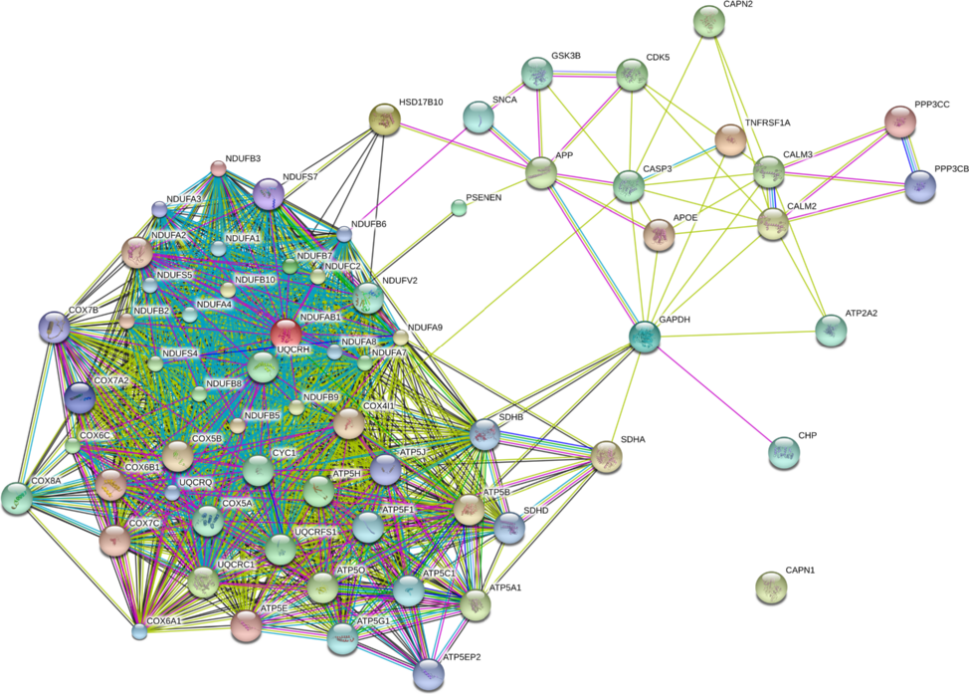
**Alzheimer-related protein association network in AD-iPS5 neurons.** Protein association network retrieved from STRING v9 using genes from the Alzheimer disease pathway down-regulated in the AD-iPS5 neurons vs. H9 neurons comparison of one neuronal differentiation each. The network circles represent proteins. The lines between the circles show the functional association. Co-expression evidence: black, database evidence: light blue, textmining evidence: yellow, experimental evidence: purple, co-occurrence evidence: blue, neighborhood evidence: green, fusion evidence: red.

**Figure 10:**
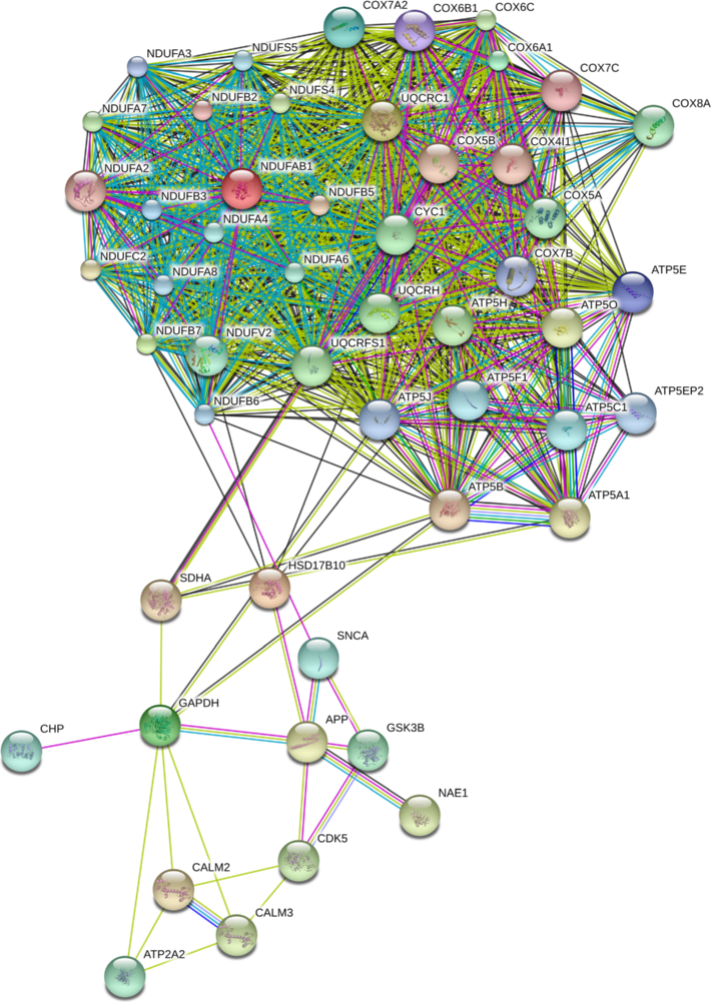
**Alzheimer-related protein association network in AD-iPS26B neurons.** Protein association network retrieved from STRING v9 using genes from the Alzheimer disease pathway down-regulated in the AD-iPS26B neurons vs. H9 neurons comparison of one neuronal differentiation each. The network circles represent proteins. The lines between the circles show the functional association. Co-expression evidence: black, database evidence: light blue, textmining evidence: yellow, experimental evidence: purple, co-occurrence evidence: blue, neighborhood evidence: green, fusion evidence: red.

**Figure 11:**
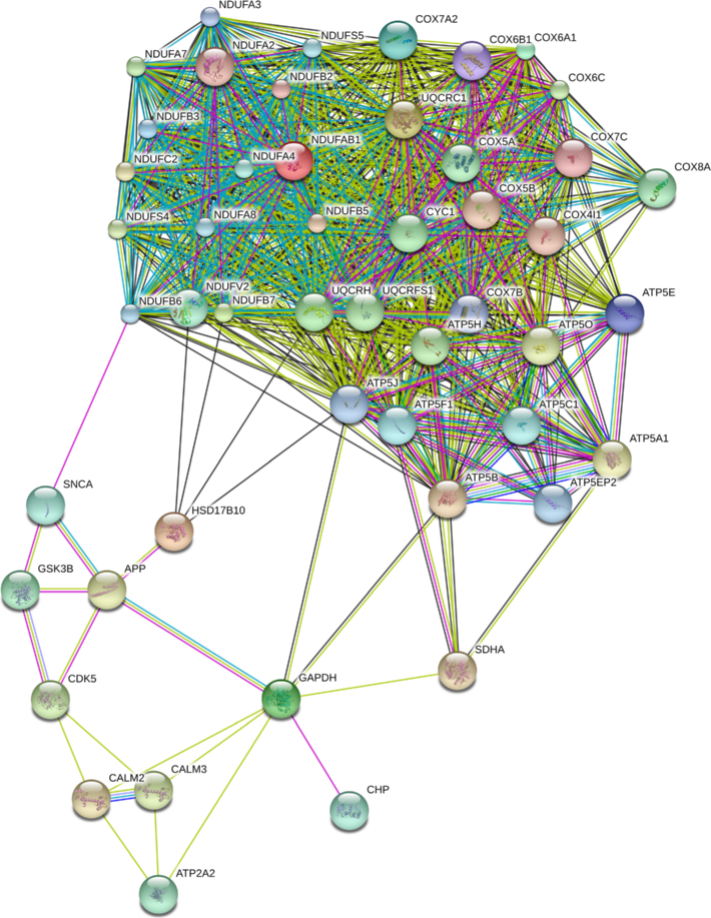
**Overlapping Alzheimer-related protein association network of AD-iPS5 neurons and of AD-iPS26B neurons.** Protein association network of genes down-regulated in both AD-iPS5 neurons vs. H9 neurons and in AD-iPS26B neurons vs. H9 neurons comparisons of one neuronal differentiation. Protein interaction network retrieved from STRING v9 using genes from the Alzheimer disease pathway down-regulated in AD-iPS5 vs. H9 neurons and in AD-iPS26B vs. H9 neurons experiments. The network circles represent proteins. The lines between the circles show the functional association. Co-expression evidence: black, database evidence: light blue, textmining evidence: yellow, experimental evidence: purple, co-occurrence evidence: blue, neighborhood evidence: green, fusion evidence: red.

To further specify the reflected Alzheimer-related phenotype in our iPSC-based neuronal disease model we constructed protein association networks by means of the gene expression data generated from one differentiation experiment. Therefore, protein-interaction networks were generated using genes annotated with AD pathway that are down-regulated in AD-iPS5 neurons vs. H9 neurons, in AD-iPS26B neurons vs. H9 neurons as well as the overlap of both datasets. We successfully modeled the association of Alzheimer-related proteins within our cellular system in both AD-iPS neuronal differentiation experiments through the subsequent comparison to non-AD embryonic stem cell line H9 neuronal cells and further network construction by applying STRINGv9. The generated networks in Figures 9, 10 and 11 depict associations between proteins in a color code. The color of the line between proteins represents the following evidence categories: neighborhood in the genome (dark green line), gene fusion (red line), co-occurrence across genomes (dark blue line), co-expression (black line), experimental/biochemical data (purple line), association in curated databases (light blue line) and co-mentioning in PubMed abstracts/textmining evidence (light green line).

The protein association networks built from genes down-regulated in AD-iPS5 vs. H9 neurons in Figure 9 contains 18 more Alzheimer-related proteins than the AD-iPS26B vs. H9 neurons network in Figure 10. These proteins are NDUFB10, NDUFA9, NDUFB8, NDUFB9, ATP5G1, CAPN2, UQCRQ, NDUFA1, CAPN1, NDUFS7, SDHB, TNFRSF1A, CASP3, APOE, SDHD, PPP3CB, PPP3CC and PSENEN (Figure 9). However, the two proteins NDUFA6 and NAE1 are only part of a network built from the AD-iPS26B vs. H9 neurons dataset (Figure 10). Interestingly, both interaction networks contain APP and GSK3Β as well as CDK5 and HSD17B10. In the AD-iPS5 vs. H9 neurons and not in the AD-iPS26B vs. H9 neurons network APP is depicted to be associated with CASP3 and APOE by experimental evidence and textmining evidence as well as with PSENEN, however, only via textmining evidence (Figure 9). In addition, only the AD-iPS26B vs. H9 neurons network depicts the association of APP and NAE1 through co-expression, database, experimental and textmining evidence (Figure 10). Furthermore, only in the AD-iPS5 vs. H9 neurons and not in the AD-iPS26B vs. H9 neurons network functional associations between CASP3 and CDK5, CAPN2, TNFRSF1A, CALM3, APOE and COX4I1 that are proven by textmining evidence could be found. TNFRSF1A which is only a part of the network in Figure 9 is associated with CDK5, CALM3 by textmining evidence and with CASP3 with additional database evidence. Exclusively in this protein association network APOE, which plays a major role in Alzheimer pathogenesis, is associated with CASP3, CALM3, CALM2 and GAPDH by textmining evidence whereas CAPN2 is associated with CASP3, CALM2 and CALM3 by textminig evidence. Surprisingly, no associations could be found for CAPN1 in this network. Additional proteins only part of the AD-iPS5 vs. H9 neurons network are PPP3CC and PPP3CB both of which are associated with each other by co-occurrence, database, experimental and textmining evidence. They are associated with CALM2 and CALM3 with experimental and textmining evidence. PSENEN, a subunit of the γ-secretase complex, occurs only in this protein association network and is associated with UQCRH via textmining and co-expression evidence. Additional proteins exclusively part of the AD-iPS5 vs. H9 neurons network are NDUFS7, NDUFA9, NDUFB8, NDUFB10, NDUFA1, NDUFB9, UQCRQ, ATP5G1, SDHB and SDHD. These are part of a complex protein association network-cluster mainly consisting of proteins involved in oxidative phosphorylation (Figure 9). Interestingly, NDUFA6 is only part of the protein association network based on the genes in the AD-iPS26B vs. H9 neurons dataset (Figure 10).

The network built from the genes overlapping between the AD-iPS5 vs. H9 neurons and AD-iPS26B vs. H9 neurons datasets in Figure 11, shows associations with experimental and textmining evidence of APP with HSD17B10, GAPDH, CDK5, GSK3B and SNCA. In addition, database evidence to prove the association of APP, SNCA and GAPDH could be found. Associations with textmining evidence between CDK5 with CALM2 and CALM3 as well as between GAPDH and ATP2A, CALM2, CALM3, SDHA, ATP5B and ATP5J could be found by our method. Furthermore, the interaction of GAPDH with ATP5B and ATP5J is associated with co-expression evidence in this network. GAPDH is associated with CHP via experimental evidence. Additional Alzheimer-related genes are interconnected to a complex protein association network cluster similar to Figures 9 and 10. consisting of proteins like NDUFB3, ATP5E, NDUFB5, UQCRC, NDUFB6, NDUFB7, ATP5B, NDUFAB1, NDUFB2 that are mainly involved in oxidative phosphorylation or in the electron transport chain in mitochondria. In our Alzheimer-related protein association network in Figure 11 we found experimental evidence association between SNCA and NDUFB6 as well as the co-expression evidence interactions of HSD17B10 with NDUFV2, NDUFB7, UQCRH and ATP5J. These associations connect Alzheimer-specific APP, GSK3B, CDK5, CALM2, CALM3 and ATP2A2 with proteins involved in Alzheimer related failure of the function of mitochondrial processes of the respiratory chain of the protein association cluster. A further association to proteins involved in oxidative phorphorylation are the interactions of GAPDH with ATP5J, ATP5B and SDHA (Figure 11).

### A subset of UPS-related genes is down-regulated in AD-iPSC neurons compared to H9 neurons

**Figure 12:**
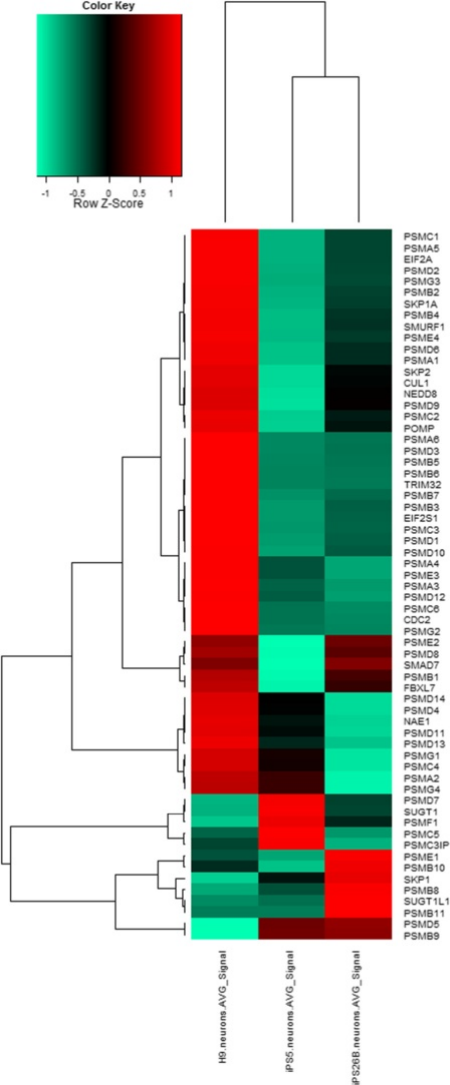
**UPS-associated gene expression in AD-iPSC neurons.** Cluster analysis based on Pearson correlation of UPS-related genes in AD-iPS neurons and H9 neurons. Up and down-regulated transcripts are depicted in red and green, respectively. RNA from AD-iPS5 neurons, AD-iPS26B neurons and H9 neurons of one well of one neuronal differentiation each was hybridized onto an Illumina human-8 BeadChip version 3. Known proteasome-related genes were selected from the microarray experiments of AD-iPS5 neurons, AD-iPS26B neurons and H9 neurons. Their Illumina average signal intensities were transformed to a logarithmic scale (log2) and clustered with the R heatmap2 function using Pearson correlation as similarity measure.

The cluster analysis of UPS-associated genes assembled both AD-iPS neurons datasets into a cluster separated from the H9 neurons. Genes were divided into three clusters (i) 36 genes which have lower gene expression values in both AD-iPS neuron compared to H9 neurons, among them *PSMC1*, *PSMA5*, *NEDD8*. (ii) 2 genes characterized by higher expression in both AD-iPS neuron compared to H9 neurons: *PSMD5*, *PSMB9*. (iii) 25 genes the expression of which varies between the three samples. The last cluster is subdivided into (i) 5 genes with high expression values in H9 neurons and AD-iPS5 neurons but not in AD-iPS26B neurons - *PSME2*, *PSMD8,* (ii) 9 genes with high expression values in H9 neurons and AD-iPS26B neurons but not in AD-iPS5 neurons - *PSMD14*, *PSMD4,* (iii) 5 genes with a low gene expression values in H9 neurons and AD-iPS26B neurons and a high gene expression in AD-iPS-5 neurons - *PSMD4*, *SUGT1*, (iv) 6 genes with low gene expression values in H9 neurons and AD-iPS5 neurons and a high gene expression values in AD-26B neurons - *PSME1*, *PSMB10* (Figure 12).

## Discussion

Little is known about the clinical onset and course of sporadic AD due to the limited insight and access to brain-derived neuronal cells from patients afflicted with neurodegenerative diseases. Therefore, it is essential that we develop new *in-vitro*-based experimental models that may reflect affected nerves in the brain. Thus, an early diagnosis could help in this regard to treat the affected individuals effectively and to test preventive approaches of sporadic Alzheimer’s disease treatment. In recent time, several research groups independently and successfully differentiated somatic cells of AD patients directly or by iPSC-based approaches into neuronal cells, and examined them with respect to the molecular basis of disease development [22-26].

The results of these studies lead to valuable insights regarding understanding of AD molecular disease, given that these innovative and predictive patient cell models displayed the AD phenotype [22]. In our current study, we confirmed that dermal fibroblasts derived from sporadic AD patients could be induced to a pluripotent state (iPSCs) and further differentiated into neuronal cells in one reprogramming and one neuronal differentiation experiment per derived AD-iPSC clone. The transcriptomes of the derived neuronal cells were characterized by the expression of neuronal markers such as *GALC, MAP2, VAMP2, HES5, SOX2, PROM1* and AD-specific gene expression patterns when compared to control neuronal cells. We successfully generated an Alzheimer’s disease-related protein association network using detected AD-related alterations of the transcriptome. As a control we used neuronal cells generated from the female embryonic stem cell line H9 in our study. This is a very clean background, it might be argued that some of the disease associated effects we see in our model come from differences between embryonic stem cells and iPS cells in general or are related to the advanced age of the AD patient (82-years-old) whose cells were used in this study. An effect of the age-related high mutation load in the parental fibroblast compared to H9 cannot be excluded but could be significant since AD is an aged-related disease and as such one would expect a higher mutation load in patients. An age-matched control iPS cell line would have been an alternative to H9 for our study. Many iPSC-based disease models compare the effect of mutations in a disease associated gene within the same genetic background. The parental fibroblasts used for AD-iPSC generation in this study did not carry any mutations in Alzheimer-related genes which excluded the possibility to use the same genetic background in our case. Based on the fact that *FABP7* a gene involved in neuronal development [35] is not expressed in H9 neurons, one could argue that the mixture of neuronal subtypes found in the neuronal differentiation of H9 is distinct from the neuronal differentiations of AD iPSCs. Despite possible differences in neuronal differentiation efficiencies or mixture of neuronal subtypes we see altered expression of AD-related genes in two neuronal differentiations of AD patient fibroblast derived iPS cell lines compared to the neuronal differentiation of H9 used as a control.

At the protein level, the neuronal cells which we derived from sporadic AD-iPSCs in a single neuronal differentiation experiment expressed p-tau and GSK3B, both valid as neuropathological proteins. Analysis of the brain of sporadic AD patients often shows intracellular accumulation of hyperphosphorylated tau proteins, an early event preceding the appearance of NFT in AD [6]. Physiological, GSK3β is a multifunctional protein kinase that phosphorylates a variety of substrates including the tau protein, which is associated with neuronal-specific microtubules. Surprisingly, in our cell model we observed the up-regulation of both isoforms of the GSK3α and GSK3β protein as well as clear formation of abnormal p-tau (Thr 231) in AD-iPSC derived neuronal cells in comparison to their parental fibroblast cells, which expressed minute levels of GSKαβ and no tau.

In a recently published study on neuronal cells derived from AD-iPSC of familial AD, abnormal p-tau expression was not detected by western blotting, probably due to the short time scale in culture [25]. Nevertheless, another study could show increased p-tau in both familial and one sporadic AD sample, however measured by the MSD phospho tau kit [22]. In the same study high amounts of GSK3β were measured in induced neuronal cells from an AD donor, this is in accordance with the observation in our AD-iPSC derived neuronal cells.

Global gene expression analysis revealed up-regulation of AD-related pathways in AD-iPS neurons of one neuronal differentiation experiment for each AD-iPS cell line such as WNT, the lysosome signalling pathway and glutathion metabolism all of which have been shown to be altered in Alzheimer’s disease [36-38]. Interestingly, we detected altered expression of genes that are involved in the alanine, aspartate and glutamate metabolism. A very recent work showed through meta-analysis of genome-wide association studies involving 2540 Alzheimer cases that changes in glutamate metabolism are overrepresented in data from patients with AD [39].

In addition, biological processes involving response to oxygen radical and response to oxidative stress were up-regulated in our sporadic AD model. Indeed these processes are known to be up-regulated in Alzheimer’s disease [40]. Moreover, biological processes such as neurogenesis appeared to be up-regulated, underlining successful neuronal differentiation. The mixture of neuronal subtypes generated by our neuronal differentiation experiments might vary as we did not carry out subtype specific differentiations.

A cluster analysis of the expression of AD-associated genes recently published confirmed differential expression in the generated AD-iPS neurons compared to H9 neurons [34].

Even though 19 of 26 genes of the cluster analysis of Alzheimer-related genes seemed to be expressed in a similar manner in H9 neurons compared to AD-iPS neurons, the transcriptomes of AD-iPS neurons were more similar to each other than to H9 neurons despite a possible variation of neuronal subtype mixtures in the conducted neuronal differentiation experiments. Differential behavior between both AD-iPS neurons experiments and the H9 neurons experiment points to genes whose up-regulation (*PTK2B*, *PICALM*, *IL8*) or down-regulation (*HLA-DRB5*) may play a major role in development of the disease in this patient.

While *PTK2B*, *IL8* and *HLA-DRB5* are clearly involved in Alzheimer pathology [34,41,42], there are controversial studies about the involvement of PICALM in Alzheimers disease [43,44].

The genes *EXOC3L2*, *CLU*, *CR1* and *TNK1* although associated with AD were not found to be expressed in AD-iPS neurons. The gene *EXOC3L2* has been associated with late onset Alzheimer disease (LOAD) in GWAS [45]. However, it could be shown that the association is likely to be caused by the close location to *APOE* and there was found no more evidence after adjustment for *APOE* [46]. Our results showing no expression for *EXOC3L2* in AD-iPS5 and AD-iPS26B neurons are in line with that finding.

The AD-association with *TNK1* which was also not expressed significantly in our experiments is unclear as several studies report ambiguous results [47,48].

In contrast to that, differentially expressed genes in AD-iPS neurons revealed the down-regulation of pathways annotated to Alzheimer’s disease, Huntington’s disease and Parkinson’s disease. The overlap of down-regulated gene expression related to Alzheimer’s disease, Huntington’s disease and Parkinson’s disease in our AD-iPS neuronal cells in Figures 7 and 8 supports the notion of a pathological mechanism common to these three neurodegenerative diseases [49].

Sixteen Alzheimer’s disease-specific genes could be confirmed to be down-regulated in AD-iPS5 (Figure 7) and 10 genes in AD-iPS26B compared to H9 neurons (Figure 8). These genes could be allocated to brain regions which are affected by Alzheimer’s disease depicted in Figure 4b and c. In both AD-iPS neuron experiments the largest number of Alzheimer-related genes was allocated to pons. Indeed, pons has been reported to have a smaller volume in patients with familial Alzheimer’s disease [50]. The second largest gene set was allocated to the brain region globus pallidus for both AD-iPS neuronal cells, a brain region which was reported to be involved in Alzheimers disease [51]. Finally, the medulla oblongata and amygdala brain regions which show deregulated gene expression in AD iPS neurons in our model were reported to be affected in AD [52,53].

We could successfully generate a protein association network consisting of AD-specific genes down-regulated in AD-iPS neurons compared to H9 neurons. The networks reflects the differences between the two AD-iPS neurons as there are more Alzheimer risk genes (for example, PSEN and APOE) present in the network built from AD-related genes down-regulated in AD-iPS5 neurons against healthy H9 neurons. Despite these differences the construction of a STRING-based protein association network representing the AD phenotype was possible with our approach for both AD-iPS neuron cell populations. APP, a protein which plays a central role in the pathology of Alzheimer’s disease, is part of the protein association network in both AD-iPS neuron cultures as well as in the association network built from the overlap of both experiments. Several studies report protein interaction networks characterizing the molecular disease phenotype in post mortem brains of sporadic AD patients or patients with familial Alzheimer disease [54,55]. Despite the usefulness of these networks for gaining insights into molecular changes in the final stage of AD, no information can be drawn about the early molecular pathology of AD with this approach. Generating iPS from AD patients in the early stage of the disease would allow modeling disease-specific changes in the AD-related protein association network over time, which provides valuable information of the development of new therapy approaches at early stages. So far, our study is the first demonstration of a protein interaction network of AD-iPS neurons derived from skin cells from an 82-year-old sporadic AD patient. Modeling sporadic AD using iPS technology as presented here enables us to formulate hypotheses to increase our understanding of AD pathogenetic mechanisms and test them by monitoring the effect on the protein association network.

Next to differential gene expression of AD-specific genes in AD-iPS derived neurons we detected the down-reglation of genes in AD-iPS neuronal cells that play a major role in the UPS. Our data revealed that the majority of the UPS-related genes are down-regulated in AD-iPS neurons compared to H9 neurons, suggesting that UPS functionality is lowered in our AD-iPS neurons but not in healthy H9 neurons. Indeed, UPS deficiency has been associated with AD pathology [18]. These results suggest that our Alzeimer model very likely reflects UPS-related features of AD pathology which are most probably present in the neuronal cells of the sporadic AD patients.

Furthermore, the UPS-related gene expression data suggests that both the constitutive and inducible proteasome play a role in AD pathology. This is reflected in the lower gene expression of the main constitutive subunits of the proteasome *PSMB5/6/7* in iPS-derived neuronal cells from the AD patient compared to H9 neurons and the higher gene expression of *PSMB9* in both AD-iPS neurons compared to H9 neurons. The genes *PSMB8* and *PSMB10,* the expression of which is higher than in H9 neurons only in the case of AD-iPS26B neurons are probably less important in AD-related pathology driven by the UPS as their expression showed a higher variation. Indeed, constitutive proteolytic activities have been reported to be decreased in AD brains, meanwhile the composition of the proteasome complex is not affected [56]. Interestingly, in contrast to the constitutive proteolytically-active subunits, the inducible ones have been reported to be highly expressed in the hippocampus (HC) of severe diseased AD patients (Braak stage ≥ III) [57,58].

In addition, we found that the expression of the gene *NEDD8* is down-regulated in AD-iPS neurons compared to the control. It is known that NEDD8 plays a role in AD pathology. The APP binding protein-1 (APP-BP1) is also increased in the AD-affected HC [16]. APP-BP1 associates with UBA3 resulting in an E1-like activating enzyme for the process of NEDD8, an ubiquitin-like protein [16]. However, NEDD8 was additionally found to be present in high amounts in neurofibrillary tangles (NFTs) and senile plaques from a patient with AD [59,60], which would suggest that NEDD8 is up-regulated in AD, which we do not see in our iPS-based model. Our data suggests that UPS dysfunction may occur early in AD pathogenesis eventually leading to cellular protein aggregates later on.

Using our neuronal cell model, we provide a proof of principle that neuronal cells differentiated from patient dermal fibroblasts-derived iPS cells offer a valuable tool for modeling early molecular pathology of AD, screening and development of appropriate drugs for the treatment of AD in the future. However, our results suggest that the different iPSC clones even derived from the same individual may give rise to different responses which is reflected in the differences between the two protein association networks generated from the gene expression data of the two different AD-iPS clones differentiated to neuronal cells in one experiment each. In our study we generated two sporadic AD-iPSC lines: one (AD-iPS26B) exhibited complete teratoma formation and normal karyotype whilst the other cell line (AD-iPS5) showed karyotype abnormalities and failed to differentiate into endoderm *in-vivo*. Due to the fact that the teratoma assay is not standardized [29] the trend now is towards a transcriptome-based classification of pluripotency (PluriTest) rather than the traditional teratoma-based assay [61]. Interestingly, only AD-iPS26B responded to the **γ**-secretase-inhibitor CE treatment in one inhibitor treatment experiment. This would suggest that the selection of iPSC clones is critical to enable the generation of results that are clinically relevant. However, neurons from AD-iPS5 showed a higher number of AD-related genes that are deregulated compared to AD-iPS26B neurons as reflected in the different protein association networks built from these two neuronal cell differentiation experiments. Therefore, the failure to respond to CE could be a reflection of this difference in AD-related gene expression between the two clones. Our findings, although based on a limited number of AD-iPSCs, have highlighted the fact that molecular pathology of sporadic Alzheimer can be modelled in disease-related protein association networks by means of iPSC technology and transcriptome analysis. Abnormalities of the karyotype in the parental AD-iPS cells should be avoided but as in our case do not necessarily lead to a distortion of gene expression-based AD protein association networks with this approach.

## Conclusion

In summary, we have generated patient-specific pluripotent stem cells from skin fibroblasts of an 82-year-old woman suffering from sporadic AD and induced these to differentiate into neuronal cells. The patient-derived cells recapitulated key features of the disease, including the expression of p-tau, GSK3β, down-regulation of AD-related genes and altered biological features caused by differential expression of genes involved in e.g. the UPS or response to oxidative stress.

The majority of the UPS genes are down-regulated in AD neurons, thus supporting the idea that dysfunctions in this system may occur early in AD pathogenesis and then lead to cellular protein aggregates at later stages of the disease. Additionally, a few genes were distinctly up-regulated in neurons derived from the two AD-iPSC lines. This may suggest that these genes are less important for AD pathogenesis as their expression varies among different neuronal lines.

In essence, we have successfully generated an Alzheimer-related protein association network using characteristic gene expression patterns detected in AD-iPSCs compared to a healthy control of one neuronal differentiation experiment. Our results lend further support to the fact that neuronal cells differentiated from iPSCs from sporadic AD patients in part recapitulate the neuropathological processes of the disease. We anticipate that iPSC-based modeling of AD as demonstrated here can be useful for formulating testable hypotheses that might eventually enhance our meager knowledge of the molecular basis of its progression and should eventually lead to the development of new drugs to prevent or treat this disease.

## Methods

### Ethics statement

Full-thickness skin biopsy was resected from the forearm of the patient undergoing surgery. The Charité University Medicine Berlin ethics committee specifically approved this study. Ethical agreement was preliminarily obtained from the guardian of the participant including written informed consent.

### Cell culture

Adult dermal fibroblasts from an 82-year-old woman (NFH-46) suffering from late-stage AD under no medication and with no family history were obtained from 6 mm full-thickness skin biopsies originating from the sun-protected forearm inner side. The skin specimens were incubated in dispase solution (2.4 U/ml) overnight at 4°C. After separation of epidermis, primary dermal fibroblasts were isolated from dermis by enzymatic digestion and were expanded within 4 weeks. The dermal fibroblasts were cultured in DMEM supplemented with 10% fetal calf serum (FCS), nonessential amino acids, L-glutamine, penicillin/streptomycin and sodium pyruvate (all from Invitrogen, Carlsbad, CA). The human embryonic stem cell (hESC) lines H1 and H9 were purchased from WiCell, Madison, WI (#WA01 and #WA09, respectively). Control iPSCs (OiPS3 and OiPS6) have recently been generated from the skin of an 84-year-old female (NFH-2 fibroblasts) [62]. hESCs and iPSCs were cultured in hESCs media containing KO-DMEM supplemented with 20% knockout serum replacement, nonessential amino acids, L-glutamine, penicillin/streptomycin, sodium pyruvate, 0.1 mM β-mercaptoethanol (all from Invitrogen) and 8 ng/ml basic fibroblast growth factor (bFGF) (Preprotech; Rocky Hill, NJ). Cultures were maintained on mitomycin C-inactivated mouse embryonic fibroblasts (MEFs) and passaged manually. For experiments feeder layer-free iPSCs and hESCs were grown on dishes coated with Matrigel (BD; San Diego, CA) in MEF-conditioned media (CM). All cultures were kept in a humidified atmosphere of 5% CO2 at 37°C. Experiments were carried out with primary fibroblasts at passages 3–6 and AD-iPS cells at passages 8–18.

### Retroviral transduction into fibroblasts and derivation of iPSCs

NFH46-derived iPSCs (AD-iPS5 and AD-iPS26B) were obtained using the Yamanaka retroviral cocktail [27] in one reprogramming experiment. Briefly, pMX vector-based OCT4, KLF4, SOX2 and c-MYC retroviruses were generated using 293 T cells, according to the conventional CaCl_2_ transfection protocol. 200,000 fibroblasts were used as input for reprogramming experiments and seeded into six wells of a six well plate. Four weeks after transduction, hESC-like colonies were manually picked and expanded for characterization as previously described [27,28].

### DNA fingerprinting analysis

In order to confirm somatic origin and to exclude cross reaction with hESCs, DNA fingerprinting analysis was performed as previously described [28]. 100 ng of genomic DNA isolated from one well per AD-iPS cell line were used for PCR amplification, following this program: 94°C for 1 min, 55°C for 1 min, 72°C for 1 min, for 40 cycles, using Dyad thermal cycler (BioRad, Hercules, CA). PCR products were resolved in 2.8% agarose gels to examine the differential amplicon mobility for each primer set: D7S796, repeat (GATA)n, average heterozygosity = 0.95. The primer sequences are listed in Additional file 11.

### Quantitative real-time polymerase chain reaction

Quantitative real-time polymerase chain reaction based gene expression analysis of pluripotency-associated and neural genes was carried out using the ABI PRISM SDS 2.1 software (Applied Biosystems, Foster City, CA) and Microsoft Excel [63]. The primer sequences are listed in Additional file 11. The data are presented as relative gene expression based on the ΔΔCt calculation over NFH-46 (Additional file 4) or adult brain tissue (Figure 4) with respect to standard error of mean (SEM). The real time PCR analysis was performed using triplicates for each repetition (n = 3). Real time PCR to confirm pluripotency gene expression was performed on 3 independent wells of AD-iPS5 and AD-iPS26B respectively. Each iPS line was split from one well into the three wells, and expanded, prior to RNA isolation. The RNA samples were not pooled. The real time PCR confirming the expression of neuronal markers was performed using cRNA derived from RNA of one neuronal differentiation of a single well of AD-iPS 5, AD-iPS 26B and H9 respectively.

### Confirmation of functional pluripotency *in-vitro*

For *in-vitro* differentiation, embryoid bodies (EBs) were generated from AD-iPSCs in one differentiation experiment each by cell harvesting and seeding onto low-attachment dishes in DMEM supplemented with 10% FCS, nonessential amino acids, L-glutamine, penicillin/streptomycin and sodium pyruvate (all from Invitrogen) without bFGF supplementation. One week later, EBs were plated onto gelatin-coated tissue culture dishes, grown for additional ten days, and analyzed by immunofluorescence-based detection of the expression of germ layer-specific proteins.

### Confirmation of functional pluripotency *in vivo*

*In-vivo* teratoma assays were performed by EPO-Berlin GmbH, Berlin-Buch, Germany. AD-iPSCs were collected by trypsinization, washed and injected s.c. into NOD.Cg-Prkdcscid Il2rgtm1Wjl/SzJ mice, commonly known as NOD scid gamma (NSG). Histological analysis was performed at the Institute for Animal Pathology, Berlin, Germany.

### Neuronal differentiation

Pluripotent stem cells (iPSCs and hESCs) were mechanically dissociated and grown in MEF-conditioned medium (CM) on matrigel-coated dishes for 72 h. Induction of neuronal cells was performed by adding 10 μM SB431542 (SB, TGFβ receptor inhibitor) and 1 μM PD0325901 (PD, MEK1/2 inhibitor) (both from Sigma-Aldrich, Deisenhofen, Germany) in the absence of bFGF [30]. The cells were grown for additional four weeks under daily medium change. The obtained neuronal cells were fixed for immunostaining or used for drug treatment. The neuronal differentiation and subsequent inhibitor treatment were carried out once per AD-iPS cell line and for the control H9.

### Alkaline phosphatase analysis and immunofluorescence staining

Alkaline phosphatase (AP) activity was visualized by the commercial AP staining kit (Millipore #SCR004; Schwalbach, Germany) according to the manufacturer’s instructions. In order to characterize pluripotency of all AD-iPSC colonies, the cells were fixed in phosphate-buffered saline (PBS) containing 4% paraformaldehyde (Science; Munich, Germany) for 20 min at room temperature, subsequently washed twice with PBS without Ca2+ and Mg2+, blocked with 10% FCS serum (Vector; Loerrach, Germany) and 0.1% Triton X-100 (Sigma-Aldrich, Germany) in PBS and proceeded to immunocytochemistry with primary antibodies against OCT4, SOX-2, KLF-4, SSEA1, SSEA4, TRA-1-60 and TRA-1-81 from the hESC characterization kit (all 1:100, Millipore #SCR004), NANOG (1:100, Abcam #ab62734, Cambridge, UK), Smooth-Muscle-Actin (SMA) (1:100, Dako #M0851, Hamburg, Germany), Alpha-Fetoprotein (AFP) (1:100, Sigma-Aldrich #WH0000174M1), SOX17 (1:50, R&D #AF1924, Minneapolis, MN), PAX6 (1:300, Covance #PRB-278P, Münster, Germany), Nestin (1:200, Chemicon #MAB5326, Nürnberg, Germany), b-Tubulin III (1:1000, Sigma-Aldrich #T8660), Brachyury (T) (1:50, R&D #AF2085). Alexa-488-conjugated secondary antibodies were used (1:300, Invitrogen #A11001). Nuclei were counter-stained with DAPI (200 ng/ml, Invitrogen #H357) and visualized using the confocal microscope LSM510 (Carl Zeiss, Jena, Germany).

### Western blot

Human AD-iPSCs and their corresponding fibroblast cells were detached from cell culture dishes by incubation with AccutaseTM (Millipore). For protein extraction, cells were harvested 48 h after compound-E treatment and lysed in RIPA-buffer supplemented with complete protease and phosphatase inhibitors cocktail (Roche, Penzberg, Germany). The protein extracts were derived from one well of a six well plate of a single neuronal differentiation. Extracts were homogenized and centrifuged at 10000 × g for 10 min. SDS–polyacrylamide gel electrophoresis (PAGE) and Western blot analysis of total proteins were performed [64]. Western blots were incubated with anti-tau monoclonal antibody (1:1000, Cell Signaling #4019; Frankfurt, Germany), anti p-tau (1:200, Santa Cruz #sc-32276; Heidelberg, Germany), anti GSK3A/B (1:1000, Cell Signaling #5676), anti-p-GSK3B (1:1000, Cell Signaling #5558), anti-b-Tubulin-III (1:1000, Sigma Aldrich #T8660) anti-Nestin (1:200, Chemicon #MAB5326), anti-Actin, anti-GAPDH (1:1000, Cell Signaling #5142). Neuroblastoma cell lysates were used as positive control for p-tau detection (Santa Cruz #SC-2410). Following incubation with a peroxidase-labelled anti-rabbit and anti-mouse secondary antibody (1:5000, Dako, #p0448, #p0447), antigen–antibody complexes were detected by ECL Western blotting detection reagents (Peqlab; Erlangen, Germany) for 1 min and exposed to imaging with the Fusion-FX7 imaging system (Peqlab).

### Global gene expression analysis

Total RNA isolated from one well of one reprogramming or neuronal differentiation experiment was quality-checked by Nanodrop analysis (Nanodrop; Wilmington, DE, USA) and 500 ng were used as input. Biotin-labeled cRNA was produced using a linear amplification kit (Ambion; Austin, TX, USA). Hybridizations, washing, Cy3-streptavidin staining and scanning were performed on the Illumina BeadStation 500 platform (Illumina; San Diego, CA, USA) according to the manufacturer’s instruction. cRNA samples were hybridized onto Illumina human-8 BeadChips version 3. The following samples were hybridized in duplicate: H9, NFH-46, NFH-2, AD-iPS5, AD-iPS26B, AD-iPS5 neurons, iPS26B neurons and H9 neurons. All basic expression data analysis was carried out using the BeadStudio software 3.0. Raw data were background-subtracted and normalized using the “rank invariant” algorithm and then filtered for significant expression based on negative control beads. Gene expression results were deposited in the Gene Expression Omnibus (GEO) repositary website http://www.ncbi.nlm.nih.gov/geo/; GEO data access number: GSE42492.

### Cluster analysis of expression of Alzheimer-associated genes

Genes associated with Alzheimer in a published genome-wide association study (GWAS) [34] and known neuronal marker genes were filtered from the microarray experiments of AD-iPS5 neurons, AD-iPS26 neurons and H9 neurons from one neuronal differentiation experiment each. Illumina detection p-values were mapped to a binary scale (0 = not expressed if p-value > 0.05, 1 = expressed if p-value < = 0.05). These values were clustered via the R heatmap2 function using Euclidean distance as distance measure.

### Differences and commonalities between neural disorders Alzheimer disease, Huntington disease and Parkinson disease

Functional annotation of significantly regulated genes from the experiments AD-iPS5 neurons vs. H9 neurons and AD-iPS26 neurons vs. H9 neurons from one neuronal differentiation was performed with the DAVID tool [65,66]. We focused the analysis on Alzheimer disease-related KEGG pathways Alzheimer disease, Huntington disease and Parkinson disease. Only down-regulated genes (compared to the reference of H9 neurons) were significantly enriched in these pathways and were therefore intersected via Venn diagrams to find overlaps and differences in the iPS cells concerning these neural disorders. All disjunct sets from this analysis are comprised in an excel table.

### Analysis of brain regions associated with regulated genes

Association of genes with tissues was downloaded from the Ensembl/Biomart Human genes 75 (GRCh37.p13) GNF/Atlas organism part annotation data set accounting for gene expression in organism parts. Only brain regions were used for follow-up processing. These brain regions were mapped to genes significantly down-regulated in the AD-iPS neuron experiments which were carried out once per AD-iPS cell line and were in the KEGG Alzheimer disease pathway but neither in Huntington disease nor in Parkinson disease pathways.

### Cluster analysis of proteasome-specific genes

Genes associated with the proteasome in the literature and in the gene definition annotation were selected from the microarray experiments of AD-iPS5 neurons, AD-iPS26B neurons and H9 neurons from one neuronal differentiation each. Their Illumina average signal intensities were transformed to a logarithmic scale (log2) and clustered with the R heatmap2 function using Pearson correlation as similarity measure.

### Building of protein association networks

Differentially expressed genes were filtered from the microarray data by comparing the signal intensities of AD-iPS neurons and H9 neurons from one neuronal differentiation experiment each. Genes with signal intensity ratios below 0.8 were considered as down-regulated. The list of official symbols of these genes was used as input for gene annotation analysis using DAVID Bioinformatics Resources 6.7 [64,65]. Subsequently, a gene list with the KEGG annotation Alzheimers disease was used as input for building of the protein association networks using STRING v9.1 [67,68].

### Sequence analysis

In this study, we performed a systematic analysis of the entire coding region including flanking intron sequences of the genes *APP*, *PSEN1* and *PSEN2* by direct sequencing. The target fragments were amplified by polymerase chain reaction (PCR) using intronic primers designed from genomic sequence with the Primer 3 software. PCR products were purified by Exo/SAP digestion (Exonuclease I; New England; Beverly, MA; shrimp alkaline phosphatase; Promega, San Diego, CA, USA) and directly sequenced using ABI-PRISM BigDye® Terminator v1.1 Cycle Sequencing Kit (Applied Biosystems) and the ABI-PRISM 3730 DNA Analyzer, as described by the manufacturer. Sequences were analyzed using Mutation Surveyor software v3.24 (SoftGenetics LLC, State College, PA). The primer sequences are listed in Additional file 12.

### Measurement of telomerase activity

The enzyme activity of telomerase was determined using the TraPEZE RT Telomerase Detection Kit (Millipore #S7710) [61].

### Karyotype analysis

For detection of possible karyotype abnormalities in two AD-iPS cell lines, chromosomal analysis after GTG-banding was performed at the Human Genetic Center, Berlin, Germany. For each cell line, 25 metaphases were counted and 6 (NFH-46), 8 (AD-iPS26B) and 10 (AD-iPS5) karyograms were analyzed [28].

### Inhibition of γ-secretase

In order to test the pharmacological response capabilities of AD-derived neurons, the same cells were treated with compound E, 2S-2-{[(3,5-difluorophenyl)acetyl]amino}-N-[(3S)-1-methyl-2-oxo-5-phenyl-2,3-dihydro-1H-1,4-benzodiazepin-3-yl]propanamide (**γ**-secretase-inhibitor; Calbiochem; Darmstadt, Germany). Two distinct concentrations were used: low 10 nM and high 100 nM. The inhibitor was added to the medium every alternate day for four weeks. The treated neuronal cells were then harvested in RIPA-buffer for protein analysis. One neuronal differentiation and subsequent inhibitor treatment was conduted. Protein was isolated from one well of the respective experiment.

### Detection of HLA haplotype

In order to further characterize the NFH-46 cell line and its immunogenetic association with AD, genomic DNA was isolated with the FlexiGene DNA kit (Qiagen; Hilden, Germany). The purified DNA was then applied to genotyping of the HLA–A,B,C and DRB1 genes by PCR with sequence-specific primers (HLA DNA typing kit; Olerup; Vienna, Austria) and sequence-specific oligonucleotide hybridization (LABType HD; BmT, Meerbusch, Germany) according to the manufacturer’s instructions.

### Statistical analysis

Data were analyzed using BeadStudio and Microsoft Excel and are expressed as mean and standard deviation. Data comparisons between two groups were performed by two-tailed unpaired Student’s *t* test, and P-values ≤ 0.05 in combination with ratios outside the interval [0.8, 1.25] were considered statistically significant.

## Additional files

Additional file 1:
**Sequencing analysis of Alzheimer-related genes** ***APP, PSEN1, PSEN2.*** Representative example for DNA sequencing of APP gene exon 16 of patient NFH-46, lack of mutations Lys670Asn and Met671Leu (a) and for exon 17 lack of mutation Val717Ile (b). Additional file 2:
**Telomerase activity in Alzheimer donor-derived AD-iPS cells.** The telomerase activity was low in the somatic fibroblast cells NFH-46 from which the two AD-iPS lines AD-iPS5 and AD-iPS26B were derived. Upon induction of pluripotency, the enzyme was reactivated in both iPS lines. Human embryonic stem cell lines H1 and H9 and the telomerase positive control cells (TPC) provided by the kit served as positive controls. The minus telomerase control (MTC, only CHAPS lysis buffer), no template control (NTC, only water) and heat inactivated cell extracts served as negative controls. The standard deviation is indicated by error bars. Additional file 3:
**Alkaline phosphatase (AP) staining and DNA fingerprinting of sporadic AD-iPS cell lines.** (a): The two iPS cell lines derived from the sporadic Alzheimer fibroblasts NFH-46 were positive for the pluripotency-associated alkaline phosphatase (AP) staining. Morphologies of both AD-iPSCs are shown in low and high magnification. (b): DNA fingerprinting confirmed the somatic origin of the two AD-iPS cell lines, AD-iPS5 and AD-iPS26B, and the lack of cross-contamination with hESC lines H1 and H9. The AD-iPS cell lines were derived in one reprogramming experiment. Additional file 4:
**Pluripotency-associated genes are expressed in AD-iPS cells in a similar manner as in ESC line H1.** Quantitative real-time PCR to analyze the expression of the most common pluripotency genes in the two generated AD-iPS lines (AD-iPS5 and AD-iPS26B) and embryonic stem cell line H1. Bars indicate the RNA level normalized to β-ACTIN first and compared to gene expression of NFH-46 (plus standard error of mean SEM; n = 3). Each AD-iPS cell line was split from one well into the three wells for expansion before the RNA was isolated. The RNA samples were not pooled. Both AD iPS cell lines were generated in one reprogramming experiment. Additional file 5:
**Microarray-based gene expression profiling of AD-iPS cells, control iPS cells and related parental fibroblast cells.** (a): AD-iPS cells (AD-iPS5 and AD-iPS26B), both from one well of one reprogramming experiment, cluster with control iPS cells (OiPS3 and OiPS6) and with hESCs (H1 and H9), and are far apart from AD fibroblasts (NFH-46) and control fibroblasts (NFH-2). (b): Table showing all the Pearson correlation values r^2^ between all the single samples analyzed. For color coding, five distinct degrees of correlation are represented: red for r^2^ = 1, orange for 1 < r^2^ < 0.9, yellow for 0.9 < r^2^ < 0.8, light yellow for 0.8 < r^2^ < 0.75, and grey for r^2^ < 0.75. Additional file 6:
***In-vitro*** **differentiation of sporadic AD-iPS cell lines.** Both lines (a) AD-iPS5 and (b) AD-iPS26B could be successfully differentiated into all three embryonic germ layers *in-vitro* through an embryoid body (EB) based differentiation approach. Indicated are the expression of marker proteins specific for ectoderm, mesoderm, and endoderm. Scale bars, 100 μm. Additional file 7:
**Teratoma formation of sporadic AD-iPS cell lines.** The differentiation potential of the two AD-iPS cell lines was tested *in-vivo* with the teratoma formation assay. AD-iPS26B successfully gave rise to teratoma containing derivatives of all three germ layers. For AD-iPS5 endodermal cells could not be clearly determined. Additional file 8:
**Karyotype of sporadic AD-iPSCs.** Karyotyping analysis of the AD-reprogrammed cells was performed. AD-iPS26B exhibited a normal female karyotype, in a similar fashion to the parental fibroblast cells NFH-46. AD-iPS5 was found to harbour next to monosomy of the X chromosome, small supernumerary marker chromosoms (circle). Additional file 9:
**Pathways and biological processes significantly up-regulated in AD-iPSC neuronal cells versus neuronal cells derived from embryonic stem cells of one neuronal differentiation experiment each.**
Additional file 10:
**Pathways and biological processes significantly down-regulated in AD-iPSC neuronal cells versus neuronal cells derived from embryonic stem cells of one neuronal differentiation experiment each.**
Additional file 11:
**List of primers used for quantitative real-time PCR, DNA fingerprinting.**
Additional file 12:
**List of primers used for sequencing of** ***APP,*** ***PSEN1,*** ***PSEN2*** **in sporadic AD-patient derived fibroblasts used to derive neuronal cells.**
