# Erratum: Induced pluripotent stem cell-derived neuronal cells from a sporadic Alzheimer's disease donor as a model for investigating AD-associated gene regulatory networks

**DOI:** 10.1186/s12864-015-1537-x

**Published:** 2015-06-06

**Authors:** Amir M Hossini, Matthias Megges, Alessandro Prigione, Bjoern Lichtner, Mohammad R Toliat, Wasco Wruck, Friederike Schröter, Peter Nuernberg, Hartmut Kroll, Eugenia Makrantonaki, Christos C Zouboulis, James Adjaye

**Affiliations:** Departments of Dermatology, Venereology, Allergology and Immunology, Dessau Medical Center, 06847 Dessau, Germany; Department of Vertebrate Genomics, Molecular Embryology and Aging Group, Max Planck Institute for Molecular Genetics, 14195 Berlin, Germany; Cologne Center for Genomics (CCG), Institute for Genetics, University of Cologne, 50931 Cologne, Germany; Institute for Transfusion Medicine Dessau, Red Cross Blood Transfusion Service NSTOB, 06847 Dessau, Germany; Institute for Stem Cell Research and Regenerative Medicine, Heinrich Heine University Duesseldorf, Moorenstr. 5, 40225 Duesseldorf, Germany; Department of Biology, Chemistry and Pharmacy, Institute of Chemistry and Biochemistry, Freie Universität Berlin, Thielallee 63, 14195 Berlin, Germany; Department of Geriatric Medicine, Geriatrics Research Group, Charité Universitätsmedizin Berlin, Reinickendorfer Str. 61, 13447 Berlin, Germany; Current address: Max Delbrueck Center for Molecular Medicine (MDC), Robert Roessle Str. 10, D-13125 Berlin, Germany

**Erratum to:** BMC Genomics 2015, 16:84 doi:10.1186/s12864-015-1262-5.

Following publication of this article it was brought to our attention that the name of author; Christos C. Zouboulis was incorrectly spelt as ‘Zoubouliss’. Eugenia Makrantonaki, Christos Zouboulis and James Adjaye are equal senior authors.

ᅟ

