# Supplementary figures and images for: Induced pluripotent stem cell-derived neuronal cells from a sporadic Alzheimer’s disease donor as a model for investigating AD-associated gene regulatory networks
Source: BMC Genomics. 2015 Feb 14;16(1):84. doi: 10.1186/s12864-015-1262-5 (PMC4344782; doi:10.1186/s12864-015-1262-5)

**a**

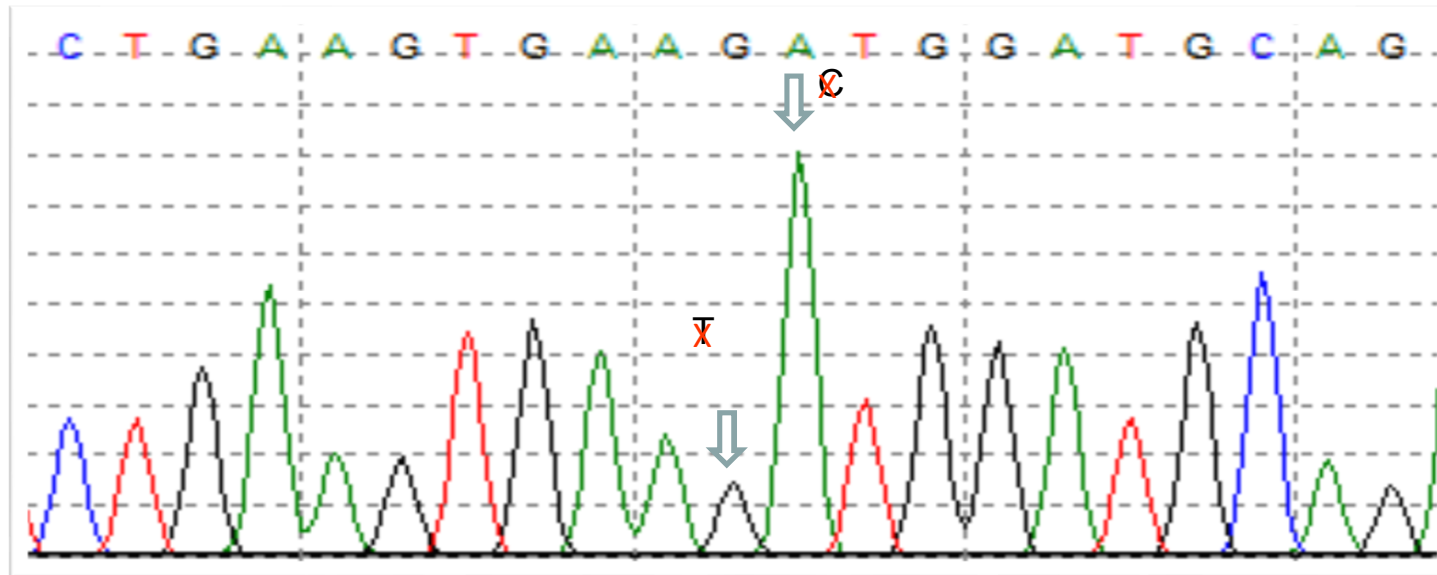

**b**

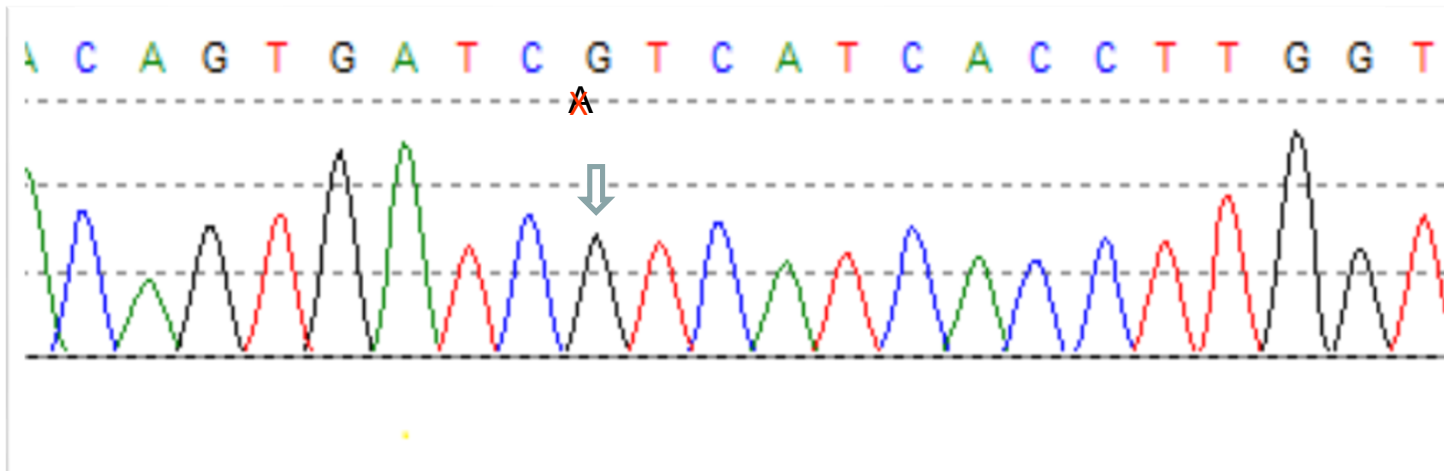

Supplement: Additional file 1: — Sequencing analysis of Alzheimer-related genes APP, PSEN1, PSEN2. Representative example for DNA sequencing of APP gene exon 16 of patient NFH-46, lack of mutations Lys670Asn and Met671Leu (a) and for exon 17 lack of mutation Val717Ile (b). [file 12864_2015_1262_MOESM1_ESM.pdf]

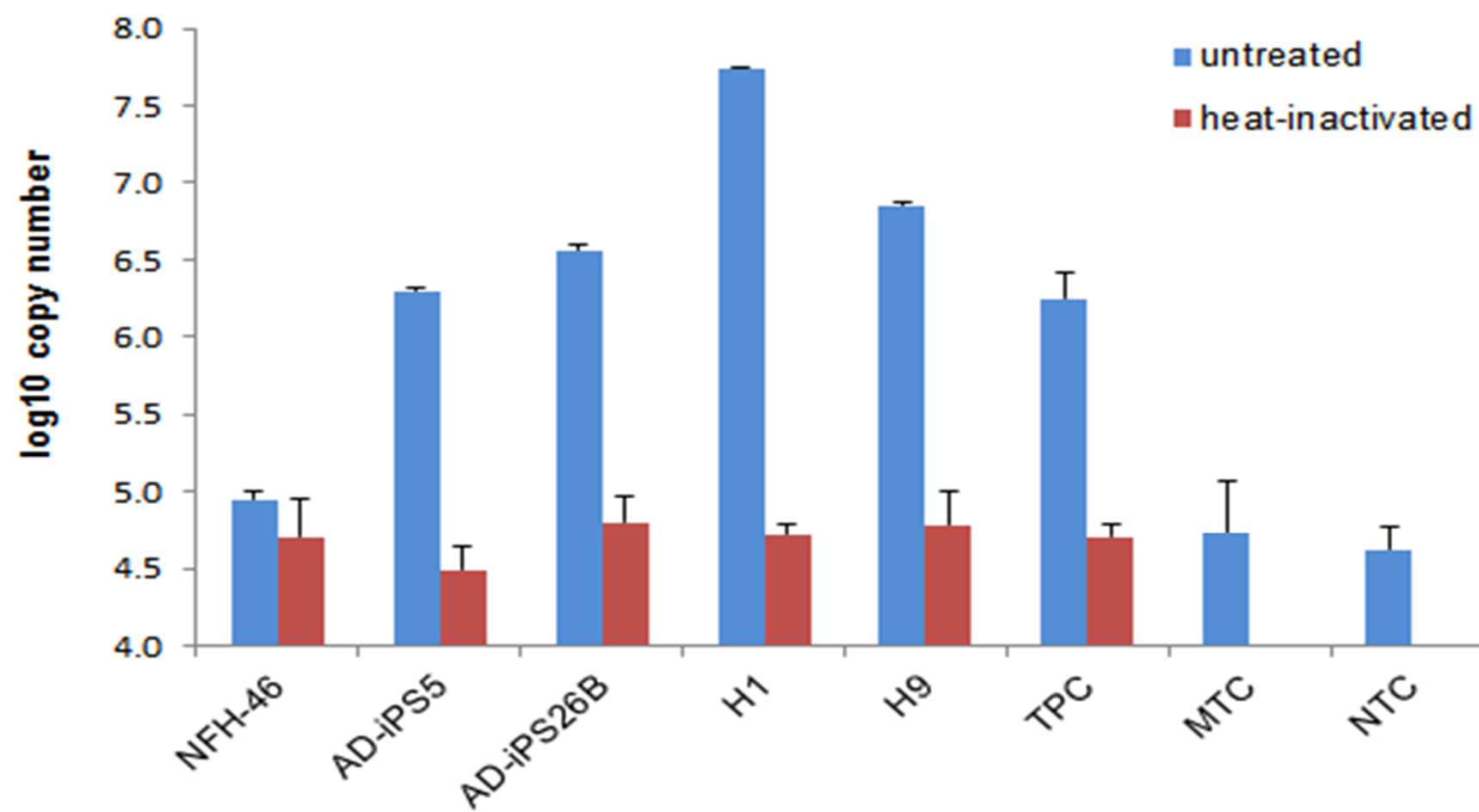

Supplement: Additional file 2: — Telomerase activity in Alzheimer donor-derived AD-iPS cells. The telomerase activity was low in the somatic fibroblast cells NFH-46 from which the two AD-iPS lines AD-iPS5 and AD-iPS26B were derived. Upon induction of pluripotency, the enzyme was reactivated in both iPS lines. Human embryonic stem cell lines H1 and H9 and the telomerase positive control cells (TPC) provided by the kit served as positive controls. The minus telomerase control (MTC, only CHAPS lysis buffer), no template control (NTC, only water) and heat inactivated cell extracts served as negative controls. The standard deviation is indicated by error bars. [file 12864_2015_1262_MOESM2_ESM.pdf]

**a**

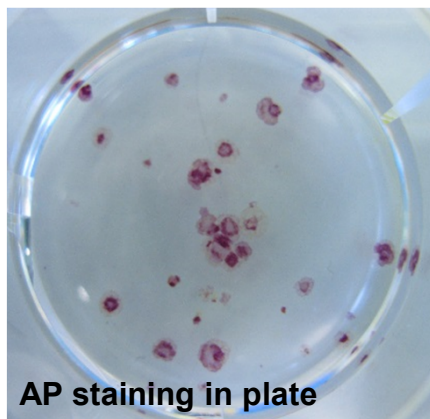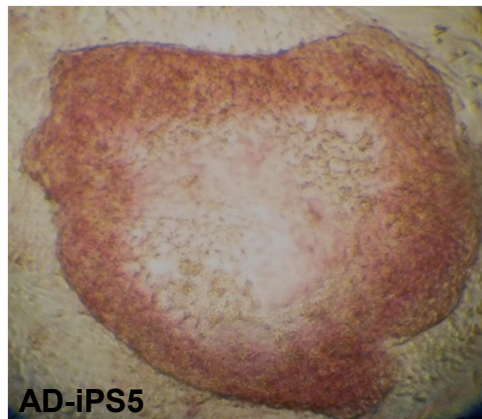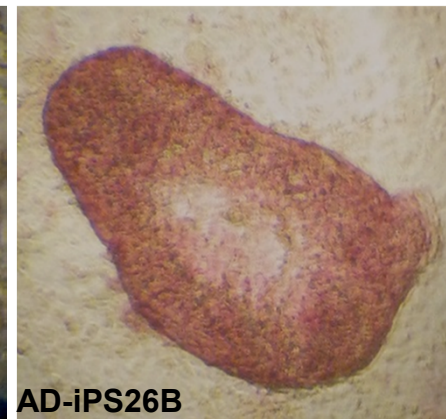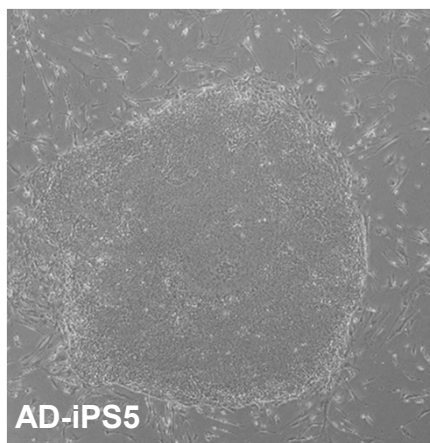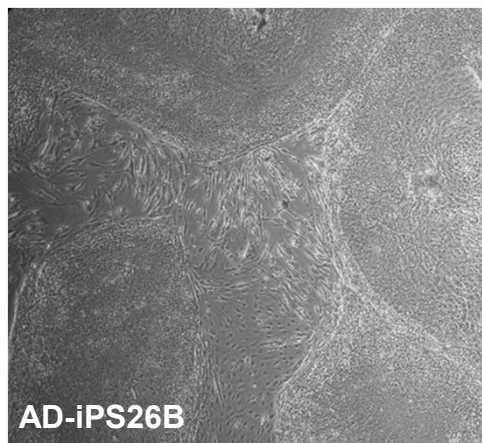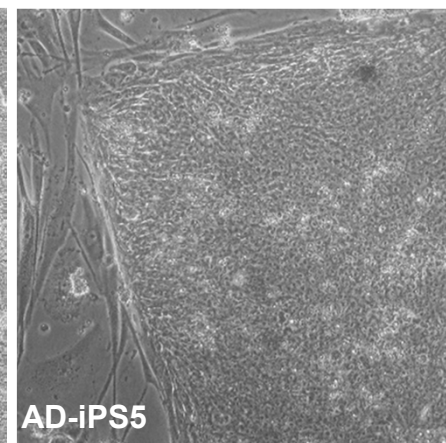

**b**

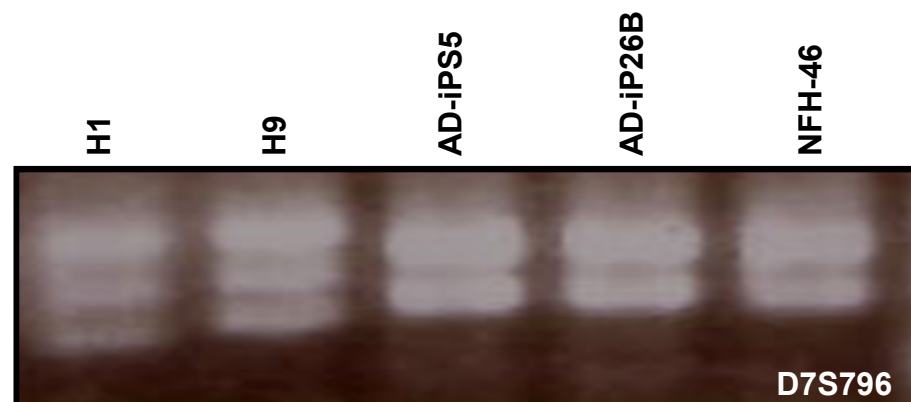

Supplement: Additional file 3: — Alkaline phosphatase (AP) staining and DNA fingerprinting of sporadic AD-iPS cell lines. (a): The two iPS cell lines derived from the sporadic Alzheimer fibroblasts NFH-46 were positive for the pluripotency-associated alkaline phosphatase (AP) staining. Morphologies of both AD-iPSCs are shown in low and high magnification. (b): DNA fingerprinting confirmed the somatic origin of the two AD-iPS cell lines, AD-iPS5 and AD-iPS26B, and the lack of cross-contamination with hESC lines H1 and H9. The AD-iPS cell lines were derived in one reprogramming experiment. [file 12864_2015_1262_MOESM3_ESM.pdf]

a

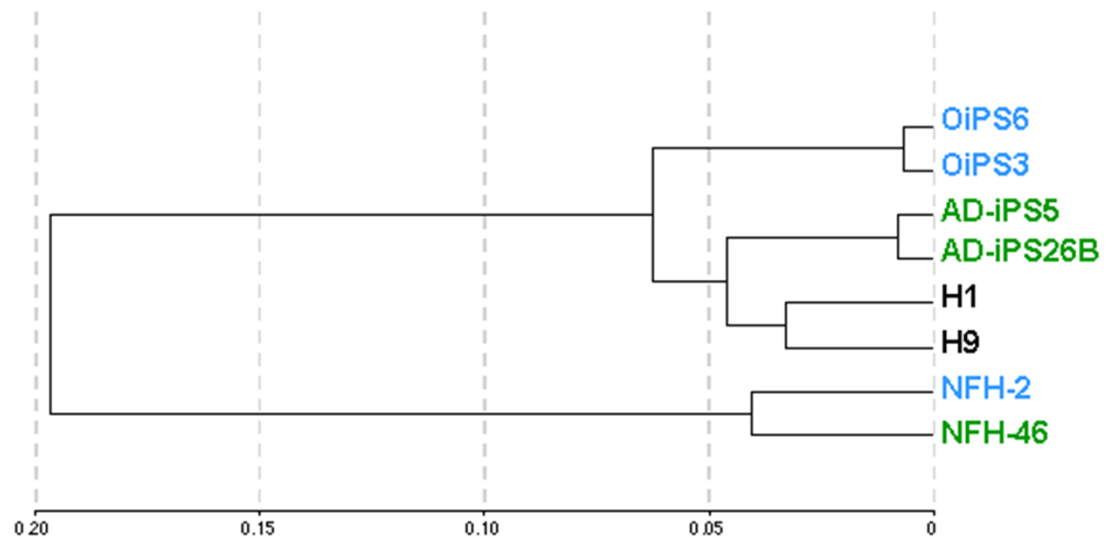

b

|           | NFH-2  | NFH-46 | OIPS3  | OIPS6  | AD-iPS5 | AD-iPS26B | H1     | H9     |
|-----------|--------|--------|--------|--------|---------|-----------|--------|--------|
| NFH-2     | 1.0000 |        |        |        |         |           |        |        |
| NFH-46    | 0.9027 | 1.0000 |        |        |         |           |        |        |
| OIPS3     | 0.6236 | 0.5967 | 1.0000 |        |         |           |        |        |
| OIPS6     | 0.6298 | 0.5789 | 0.9876 | 1.0000 |         |           |        |        |
| AD-iPS5   | 0.6456 | 0.6490 | 0.9022 | 0.8853 | 1.0000  |           |        |        |
| AD-iPS26B | 0.6757 | 0.6854 | 0.8912 | 0.8692 | 0.9844  | 1.0000    |        |        |
| H1        | 0.6088 | 0.5961 | 0.8939 | 0.8760 | 0.9219  | 0.9144    | 1.0000 |        |
| H9        | 0.6629 | 0.6442 | 0.8706 | 0.9014 | 0.9014  | 0.9043    | 0.9358 | 1.0000 |

|  |                     |
|--|---------------------|
|  | $r^2 = 1$           |
|  | $1 < r^2 < 0.90$    |
|  | $0.90 < r^2 < 0.80$ |
|  | $0.80 < r^2 < 0.75$ |
|  | $r^2 < 0.75$        |

Supplement: Additional file 5: — Microarray-based gene expression profiling of AD-iPS cells, control iPS cells and related parental fibroblast cells. (a): AD-iPS cells (AD-iPS5 and AD-iPS26B), both from one well of one reprogramming experiment, cluster with control iPS cells (OiPS3 and OiPS6) and with hESCs (H1 and H9), and are far apart from AD fibroblasts (NFH-46) and control fibroblasts (NFH-2). (b): Table showing all the Pearson correlation values r2 between all the single samples analyzed. For color coding, five distinct degrees of correlation are represented: red for r2 = 1, orange for 1 < r2 < 0.9, yellow for 0.9 < r2 < 0.8, light yellow for 0.8 < r2 < 0.75, and grey for r2 < 0.75. [file 12864_2015_1262_MOESM5_ESM.pdf]

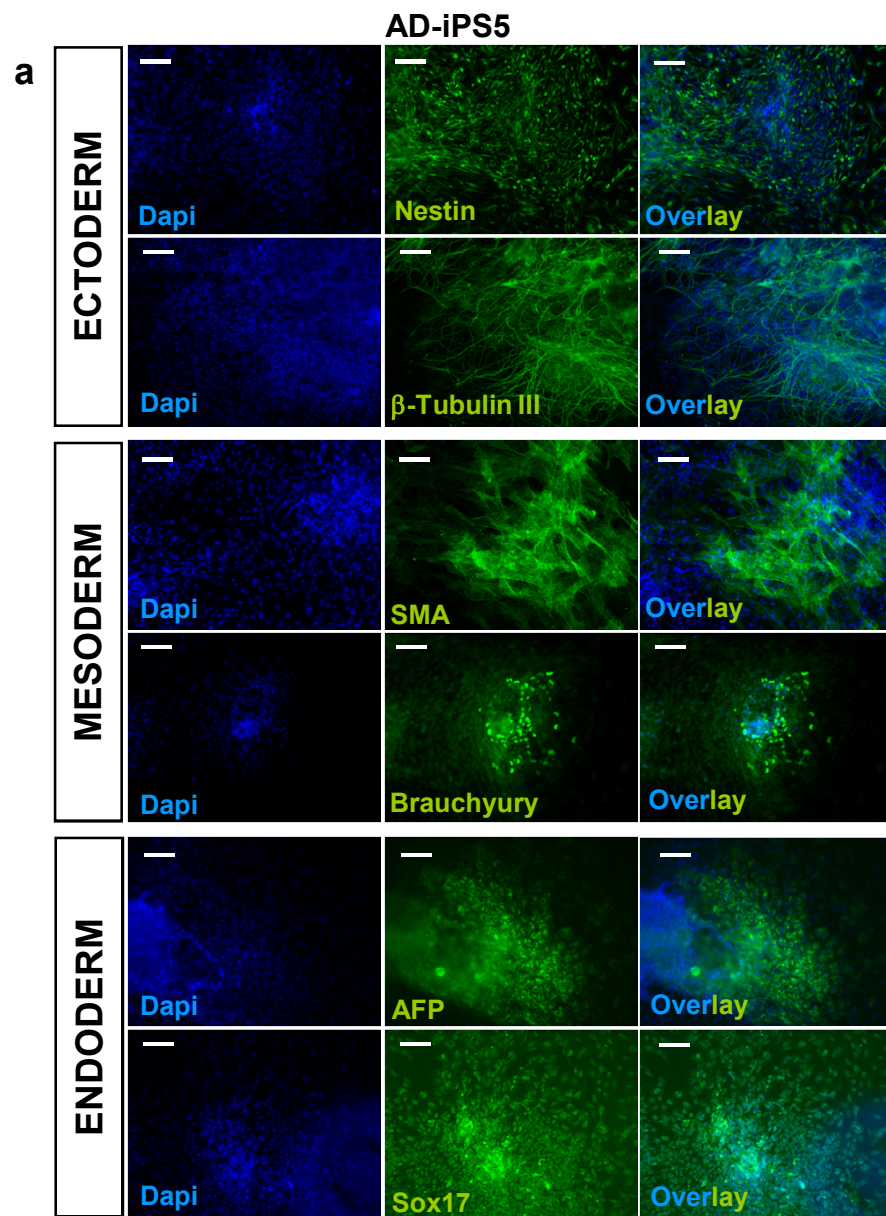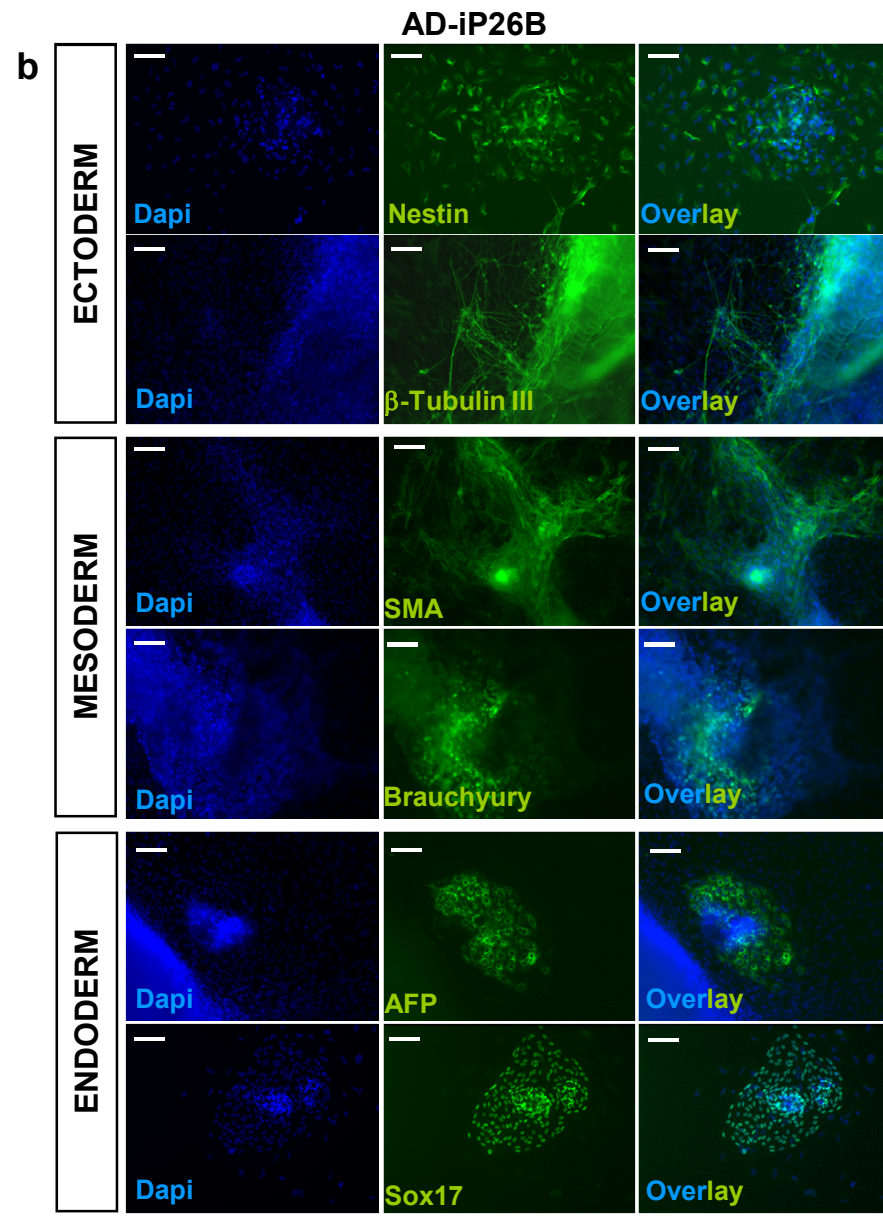

Supplement: Additional file 6: — In-vitro differentiation of sporadic AD-iPS cell lines. Both lines (a) AD-iPS5 and (b) AD-iPS26B could be successfully differentiated into all three embryonic germ layers in-vitro through an embryoid body (EB) based differentiation approach. Indicated are the expression of marker proteins specific for ectoderm, mesoderm, and endoderm. Scale bars, 100 μm. [file 12864_2015_1262_MOESM6_ESM.pdf]

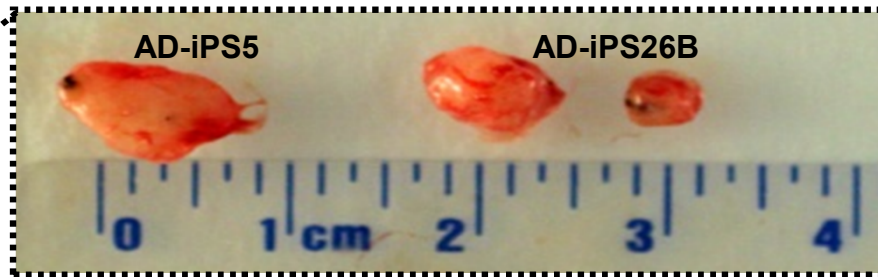

**Ectoderm, Nerves**

**Mesoderm, Cartilage**

**Endoderm, Intestine**

**AD-iPS26B**

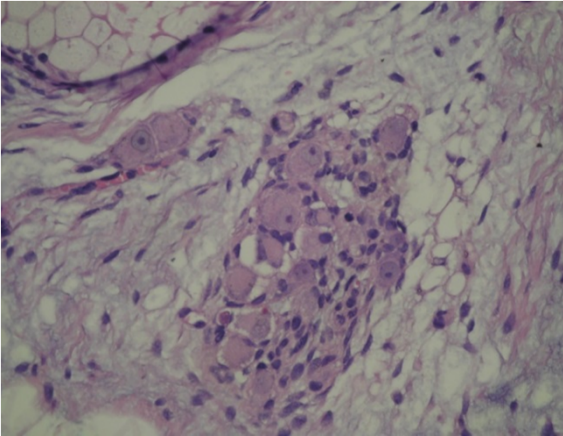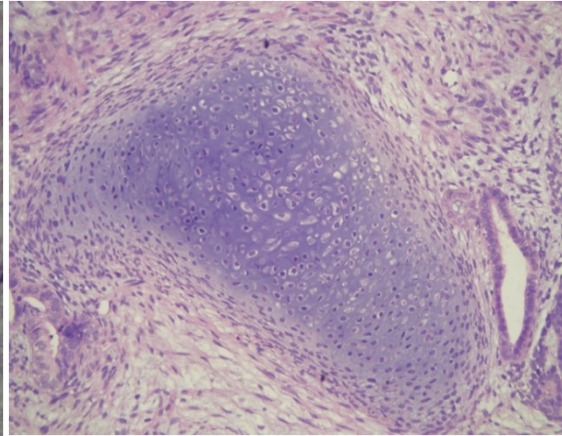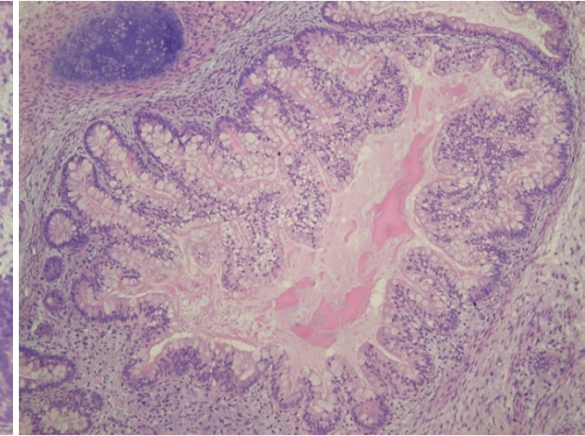

**AD-iPS5**

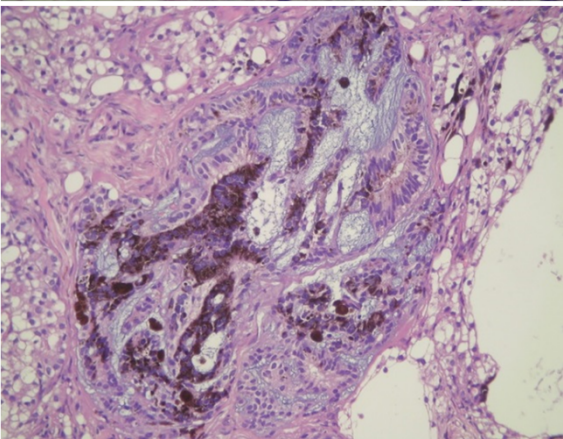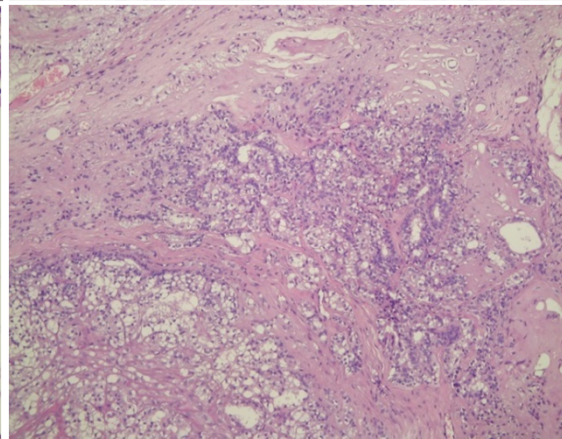

**ND**

Supplement: Additional file 7: — Teratoma formation of sporadic AD-iPS cell lines. The differentiation potential of the two AD-iPS cell lines was tested in-vivo with the teratoma formation assay. AD-iPS26B successfully gave rise to teratoma containing derivatives of all three germ layers. For AD-iPS5 endodermal cells could not be clearly determined. [file 12864_2015_1262_MOESM7_ESM.pdf]

## AD-iPS5

## NFH-46

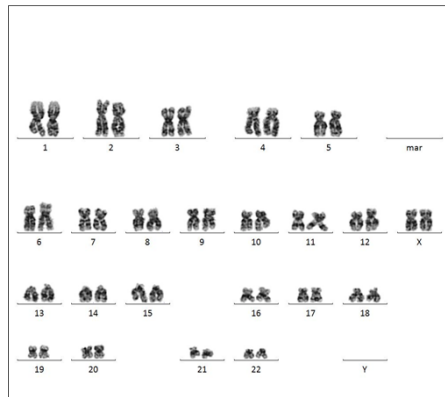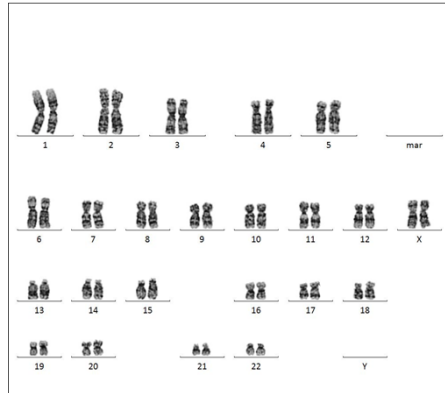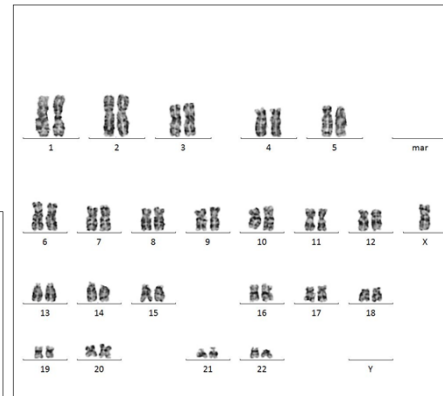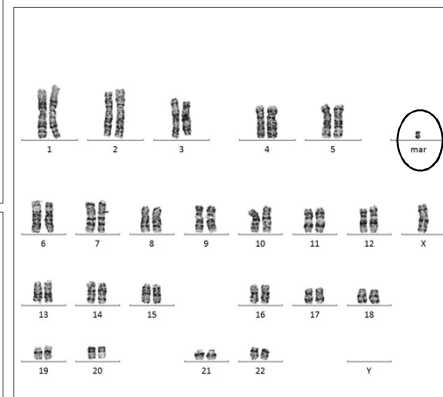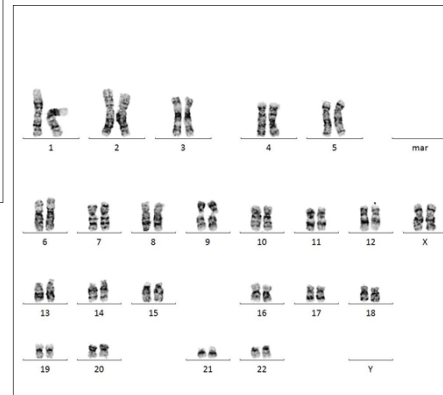

## AD-iPS26B

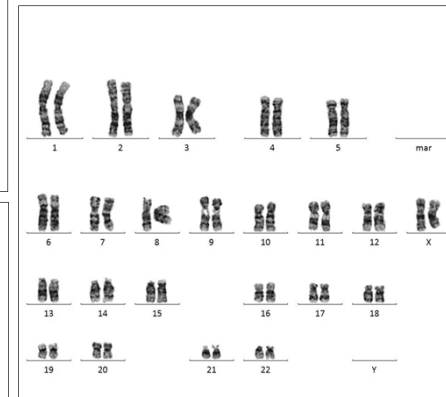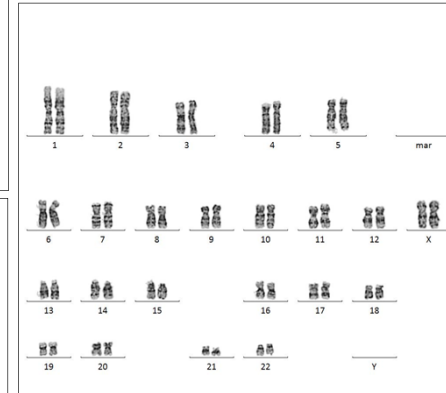

Supplement: Additional file 8: — Karyotype of sporadic AD-iPSCs. Karyotyping analysis of the AD-reprogrammed cells was performed. AD-iPS26B exhibited a normal female karyotype, in a similar fashion to the parental fibroblast cells NFH-46. AD-iPS5 was found to harbour next to monosomy of the X chromosome, small supernumerary marker chromosoms (circle). [file 12864_2015_1262_MOESM8_ESM.pdf]
