# Supplementary material for: Induced pluripotent stem cell-derived neuronal cells from a sporadic Alzheimer’s disease donor as a model for investigating AD-associated gene regulatory networks
Source: BMC Genomics. 2015 Feb 14;16(1):84. doi: 10.1186/s12864-015-1262-5 (PMC4344782; doi:10.1186/s12864-015-1262-5)
Supplement: Additional file 4: — Pluripotency-associated genes are expressed in AD-iPS cells in a similar manner as in ESC line H1. Quantitative real-time PCR to analyze the expression of the most common pluripotency genes in the two generated AD-iPS lines (AD-iPS5 and AD-iPS26B) and embryonic stem cell line H1. Bars indicate the RNA level normalized to β-ACTIN first and compared to gene expression of NFH-46 (plus standard error of mean SEM; n = 3). Each AD-iPS cell line was split from one well into the three wells for expansion before the RNA was isolated. The RNA samples were not pooled. Both AD iPS cell lines were generated in one reprogramming experiment. [file 12864_2015_1262_MOESM4_ESM.pdf]

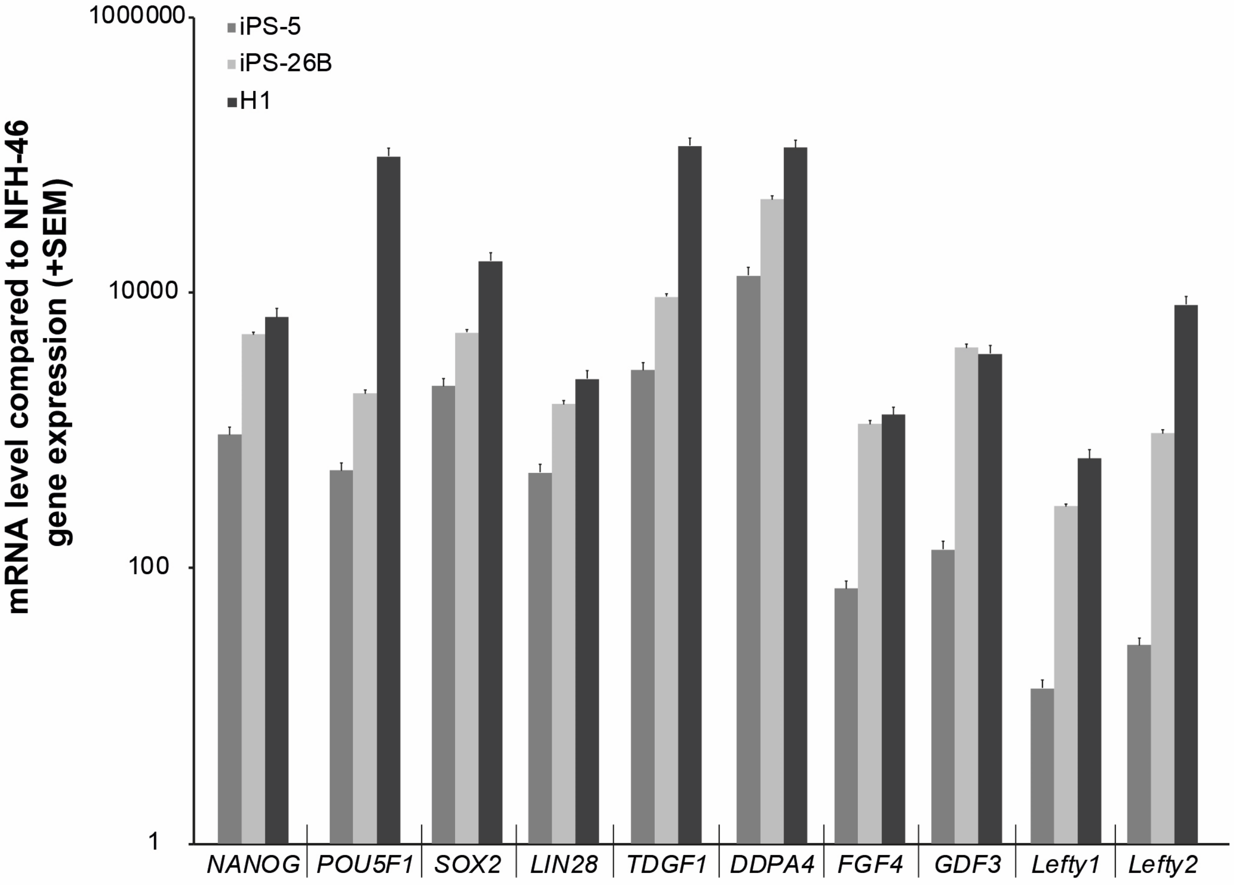

Additional_file_4: Pluripotency associated genes are expressed in AD-iPSCs in a similar fashion as in ESC line H1 Quantitative real-time PCR to analyze the expression of the most common pluripotency genes in the two generated AD-iPS lines (AD-iPS-5 and AD-iPS-26B) and embryonic stem cell line H1. Bars indicate the RNA level normalized to β-ACTIN first and compared to gene expression of NFH-46 (plus standard error of mean SEM; n=3).
